# Bayesian reconstruction of *Mycobacterium tuberculosis* transmission networks in a high incidence area over two decades in Malawi reveals associated risk factors and genomic variants

**DOI:** 10.1099/mgen.0.000361

**Published:** 2020-04-01

**Authors:** Benjamin Sobkowiak, Louis Banda, Themba Mzembe, Amelia C. Crampin, Judith R. Glynn, Taane G. Clark

**Affiliations:** ^1^​ Faculty of Infectious and Tropical Diseases, London School of Hygiene and Tropical Medicine, London, UK; ^2^​ Malawi Epidemiology and Intervention Research Unit, Malawi; ^3^​ Faculty of Epidemiology and Population Health, London School of Hygiene and Tropical Medicine, London, UK; ^4^​ Institute of Health and Wellbeing, University of Glasgow, Glasgow, UK; ^‡^​ Present address: Division of Respiratory Medicine, University of British Columbia, Vancouver, Canada, and British Columbia Centre for Disease Control, Vancouver, Canada

**Keywords:** *Mycobacterium tuberculosis*, tuberculosis, bioinformatics, molecular epidemiology, Bayesian analysis, pathogen transmission

## Abstract

Understanding host and pathogen factors that influence tuberculosis (TB) transmission can inform strategies to eliminate the spread of *Mycobacterium tuberculosis (Mtb*). Determining transmission links between cases of TB is complicated by a long and variable latency period and undiagnosed cases, although methods are improving through the application of probabilistic modelling and whole-genome sequence analysis. Using a large dataset of 1857 whole-genome sequences and comprehensive metadata from Karonga District, Malawi, over 19 years, we reconstructed *Mtb* transmission networks using a two-step Bayesian approach that identified likely infector and recipient cases, whilst robustly allowing for incomplete case sampling. We investigated demographic and pathogen genomic variation associated with transmission and clustering in our networks. We found that whilst there was a significant decrease in the proportion of infectors over time, we found higher transmissibility and large transmission clusters for lineage 2 (Beijing) strains. By performing evolutionary convergence testing (phyC) and genome-wide association analysis (GWAS) on transmitting versus non-transmitting cases, we identified six loci, *PPE54*, *accD2*, *PE_PGRS62*, *rplI*, *Rv3751* and *Rv2077c*, that were associated with transmission. This study provides a framework for reconstructing large-scale *Mtb* transmission networks. We have highlighted potential host and pathogen characteristics that were linked to increased transmission in a high-burden setting and identified genomic variants that, with validation, could inform further studies into transmissibility and TB eradication.

## Data Summary

**1**. All raw *Mycobacterium tuberculosis* sequence data are available from the European Nucleotide Archive (ENA) short-read archive (project ID ERP000436 and ERP001072).

**2**. *M. tuberculosis* strain H37Rv is available from GenBank; accession number NC_000962.3.

**Impact Statement**

Tuberculosis (TB), caused by *Mycobacterium tuberculosis *, remains a major global health concern, responsible for around 1.64 million deaths globally in 2017, with a greater disease burden in high-incidence regions. Studies detailing the epidemiological and genetic factors influencing TB transmission are complicated by the difficulty of accurately resolving accurate transmission due to a variable latency period in which within-host evolution can occur and low mutation rate. Our work reconstructs transmission networks in *M. tuberculosis* cases collected as part of a large-scale, long-term study in a high-burden region of northern Malawi, applying a sophisticated Bayesian approach that incorporates parameters for incomplete sampling of the population and within-host pathogen evolution to identify risk factors associated with recent transmission and onward infection. Our approach is of high interest to those using genomics in clinical and research settings, in particular to the tuberculosis research and clinical communities and those investigating outbreaks and pathogen transmission, where the methodological pipeline described in our study can be applied in different settings. In addition, the risk factors and genomic variants found to be associated with transmissibility in *M. tuberculosis* can inform further work on tuberculosis transmission and management for the spread of infection.

## Introduction

Establishing patterns of transmission – who infected whom – for infectious pathogens is critical for controlling outbreaks and informing health management strategies to prevent the spread of infection. Tuberculosis (TB), caused by *Mycobacterium tuberculosis (Mtb*), remains a major global health concern, responsible for 1.6 million deaths in 2017 alone, including nearly 400 000 deaths attributed to HIV-associated infection [1]. Despite initiatives aimed at reducing global incidence rates, such as the World Health Organization’s ‘End TB Strategy’ [1], many regions are falling behind set targets for case reduction. Understanding and preventing transmission is fundamental to disease control, but the accurate characterization of networks is difficult, especially as the onset of active disease follows a long and highly variable latency period during which within-host evolution can occur [2, 3]. There is a paucity of long-term studies in high-incidence areas [4–7] to assist with providing much needed biological and epidemiological insights into transmission.

The Karonga Prevention Study (now Malawi Epidemiology and Intervention Research Unit, MEIRU) is based in Karonga District in northern Malawi, which has a population of over 300 000, a high TB incidence (~100 cases per 100 000 population) and an HIV prevalence of around 10 % [6, 8]. Since the 1980s, it has been conducting research on TB in the area, collecting sputum specimens from individuals presenting at the district hospital and peripheral health centres with suspected TB [9, 10]. More than 2100 *Mtb* DNA samples from the mid-1990s onwards, encompassing strains of the major lineages 1–4 [6, 11, 12], have undergone whole-genome sequencing (WGS). These genomic data are supported by in-depth demographic data, including age, sex, previous disease history and HIV status. The combined dataset presents a unique opportunity to investigate evolutionary relationships among TB patients in a high-incidence area over an extended study period (nearly 20 years). The samples collected between 1995–2010 have previously been used to assess transmission using a simple disease outbreak model, allowing up to 10 single-nucleotide polymorphism (SNP) differences between cases [6, 13]. This simple thresholding approach revealed decreases in transmission over time and with age, as well as lineage differences in transmission [highest in lineage 2 (East Asian, including Beijing) and lowest in lineage 1 (Indo-Oceanic)] [6].

Using an SNP threshold for establishing transmission links is suitable for confirming the absence of transmission between distant strains, but can be over-simplistic for determining the timing and direction of actual transmission events in evolving populations [14]. Different thresholds have been applied previously across *Mtb* transmission studies [15–18], and the amount of genomic variation in strains can be influenced by the culturing protocol and bioinformatics pipeline used to call SNPs, making a consensus difficult. Most importantly, it does not explicitly allow for missing links in transmission chains.

This study investigated *Mtb* transmission dynamics in a high-TB-burden setting using advanced computational techniques. We applied probabilistic modelling to infer high-likelihood transmission events in 1857 TB cases from Karonga between 1995 and 2014. Specifically, the TransPhylo software package [3] was employed to provide Bayesian model-based inference of transmission reconstruction across a time-calibrated phylogenetic input, allowing for within-host diversity and incomplete sampling to infer the likelihood and time of transmission events. By accurately constructing transmission networks, we aimed to identify demographic factors that are associated with: (i) recent transmission; (ii) large, persisting transmission clusters; and (iii) transmissibility. We also applied phylogenetic convergence testing (phyC) and genome-wide association (GWAS) to detect pathogen genetic variants that were associated with a transmissible phenotype.

## Methods

### Sample collection and preparation

In Karonga District, patients exhibiting symptoms of TB were reviewed by project staff at the district hospital and local health centres, with those diagnosed with the disease interviewed to obtain further details. The information collected included: sex, age and contact with prior TB cases. Patients were HIV-tested after counselling and, if consent was given, a minimum of three sputum samples were taken from each patient. Further details of the study design are available elsewhere [8, 9]. The study was approved by the National Health Sciences Research Committee in Malawi and by the London School of Hygiene and Tropical Medicine ethics committee. Informed consent was obtained for all participants.

### Sequencing and variant calling

WGS was carried out on >2000 *Mtb* isolates from Karonga (European Nucleotide Archive at EMBL-EBI, study numbers ERP000436 and ERP001072). DNA was extracted from a sweep of multiple colonies from solid cultures and sequenced using the Illumina HiSeq 2000 platform, generating 100 bp paired-end reads. Additional culture and sequencing information has been provided elsewhere [6]. Quality checking of sequenced reads was carried out using FastQC software, with adapter sequences and low-quality reads removed using the Trimmomatic program. Reads were mapped to the *Mtb* H37Rv reference strain (GenBank no.: NC_000962.3) with BWA-mem software [19] and SNP and small insertion and deletion (INDEL) variants were called using the Samtools suite [20]. Low-quality variants (phred score Q<20, read depth DP<5) and SNPs found in repeat regions were excluded [21]. Variants were removed if there was a missing call (either through non-alignment of reads or low coverage) in >10 % of samples. Where there were multiple, longitudinal samples taken from a patient during the same disease episode of disease, the earliest collected sample was used. Samples with a high likelihood of mixed infection (two or more concurrent *Mtb* strains) were identified [12] and removed from further analysis. In the remaining 1857 samples, heterozygous sites were called as the majority allele if there was at least 75 % consensus across reads, with a minimum coverage of 20-fold; otherwise they were called as missing. Additionally, *de novo* assembly of isolates was carried out with VelvetOptimiser [22], a wrapper script that optimizes parameters for the Velvet assembler software [23] . Genomes were annotated using Prokka [24] and Roary [25].

### Phylogenetic analysis

Initially, broad putative clusters of cases were constructed for application of the Bayesian transmission inference analysis. The clusters were constructed using R software to group cases with a maximum pairwise SNP distance threshold of 50 SNPs, i.e. a case was included in a cluster if there were ≤50 SNP differences from one or more cases within this cluster. The threshold to determine potential transmission links between patients has previously typically been fixed between 5 and 12 SNP differences [6, 15, 26], although recent analysis of within-patient variation has suggested this estimate may be too low [27]. We chose to define broad initial clusters for further analysis to allow for missing cases and potential transmission between more genetically distant cases.

Phylogenetic trees were assembled for each transmission cluster with beast v1.8 [28] using SNP data (excluding highly variable *PE/PPE* and known antimicrobial resistance associated genes, and INDELs) and calibrated by episode start dates (earliest of specimen collection or registration) at the tips. Prior parameters were optimized for each cluster tree by performing preliminary analyses with 5×10^7^ Markov chain Monte Carlo (MCMC) iterations to determine the substitution model [Hasegawa, Kishino and Yano (HKY) Gamma; generalized time-reversible (GTR) Gamma]; the molecular clock (strict; uncorrelated relaxed); and the population size and growth (constant; exponential; extended Bayesian skyline plot). The performance of each prior parameter iteration was assessed through the comparison of posterior marginal likelihood estimates and checked for convergence using traces of effective sample size (ESS) distributions in Tracer v1.8 software. Three final runs of 10^8^ MCMC iterations using the best fitting prior parameters were combined using LogCombiner v1.8, discarding a 10 % burn-in for each run, to reach posterior ESS distributions above 200 for each parameter. Final maximum clade credibility trees summarizing the posterior sample of trees in the combined MCMC runs were produced using TreeAnnotator v.1.8 software. Additionally, population-level and lineage-specific maximum-likelihood (ML) phylogenies were constructed with RAxML [29] using the GTR+GAMMA substitution model and 1000 bootstrap replicates.

### Inferring transmission events

We used the R software package TransPhylo [3] to reconstruct transmission within our clusters, allowing for unsampled cases and within-host diversity. The program employs a stochastic branching process algorithm using the tip-calibrated phylogenetic trees as input [28], incorporating information such as branch lengths and substitution rates. The algorithm requires the user to define model priors estimated from characteristics of the pathogen and sample population, such as generation time and sampling density, which we tested through a sensitivity analysis on two large clusters representing lineage 2 (*n*=27) and lineage 3 (*n*=37) strains. The results of the sensitivity analysis and more detailed explanations of the rationale behind these prior parameter choices are specified in Supplementary Material S2.

The generation time is defined as the time between a host becoming infected and infecting another individual, modelled as a gamma distribution. This parameter is particularly important to define for pathogens with potentially long, variable periods of latency, such as *Mtb*, where the time between a host becoming infected and infecting others can range from a few weeks to many years [2]. Due to this highly variable latent and infectious period in *Mtb* infections, we set a wide generation time gamma distribution with a median of 3.9 years and a long tail (gamma shape=2.2, scale=2.1, rate=0.48).

Prior values for the sampling interval (the time between the onset of infection and the sample collection date, modelled with a gamma distribution), sampling density (*π*), basic reproductive number (*R*) and within-host effective population size (*N*_eg_ *),* were also specified to refine the biological relevance of the model. The sampling interval prior distribution was chosen to be the same as the generation time (median of ~3.9 years; gamma shape=2.3, scale=2.1) as the time from diagnosis to end of infectiousness is short compared to the latent period (Supplementary Material S2). We set *R=*1.75 [estimated from the sensitivity analysis (Supplementary Material S2)] and *N*_eg_=1.48 (based on a previous study [14]), with these two values being updated through MCMC iterations. Finally, we chose to test a fixed sampling density of *π*=0.5 based on the results of sensitivity analysis and allowing for unsampled cases through the unavailability of WGS data or undiagnosed cases.

The algorithm was applied to each cluster for 5×10^5^ MCMC iterations, sampling every 10 000 states, with a 10 % burn-in, and convergence-tested through inspection of trace plots of effective sample size (ESS). Transmission links with a posterior probability of more than 0.5 were accepted and the direction was inferred by using the highest probability where links in both directions were accepted. Where more than one potential infector case was inferred for the same recipient, the transmission link with the highest probability was taken. We accounted for the evidence that transmission from patients with extrapulmonary TB is highly unlikely [30] by excluding these links. Manual inspection of small INDEL profiles between linked cases and tracing of epidemiological links between cases (where available) were used to confirm transmission links and the direction of transmission.

### Transmission networks and demographic associations

We characterized the final set of transmission networks as including cases either directly linked or with up to two unsampled hosts between the transmission events inferred through the TransPhylo analysis. The host demographic and epidemiological metadata collected with each case were analysed for significant associations with recent infection (transmission within previous 3 years, first 3 years of data collection excluded), large clusters (≥10 sampled cases and ≥1 case per year) and transmissibility (cases transmitting to a recipient within 4 years versus single cases, last 4 years of data collection excluded), through logistic regression. These metadata included major lineage, age, sex, collection date, HIV status, treatment outcome, birthplace, recent residence, and isoniazid and rifampicin resistance.

### Genomic variant analysis

SNPs associated with transmissibility were identified by applying phyC, a test for detecting shared variants among groups through evolutionary convergence [31], comparing infector TB cases (cases transmitting to a recipient within 4 years) against the cases not found in a transmission cluster. We tested for sites that were associated with transmissibility in separate lineages and across the whole population. The likelihood of association was established by significance testing with Fisher’s exact test (*P*<0.05 [31]). A complementary mixed model GWAS approach [32] was also conducted to reveal any SNPs or loci highly associated with transmitting cases, including an analysis of aggregated numbers of non-synonymous mutations per gene. A significance threshold of *P*<10^−5^ was assigned by applying a Bonferroni correction for multiple testing. All analyses were performed in *R* and Python.

## Results

### *Mtb* lineages

**Fig. 1.**
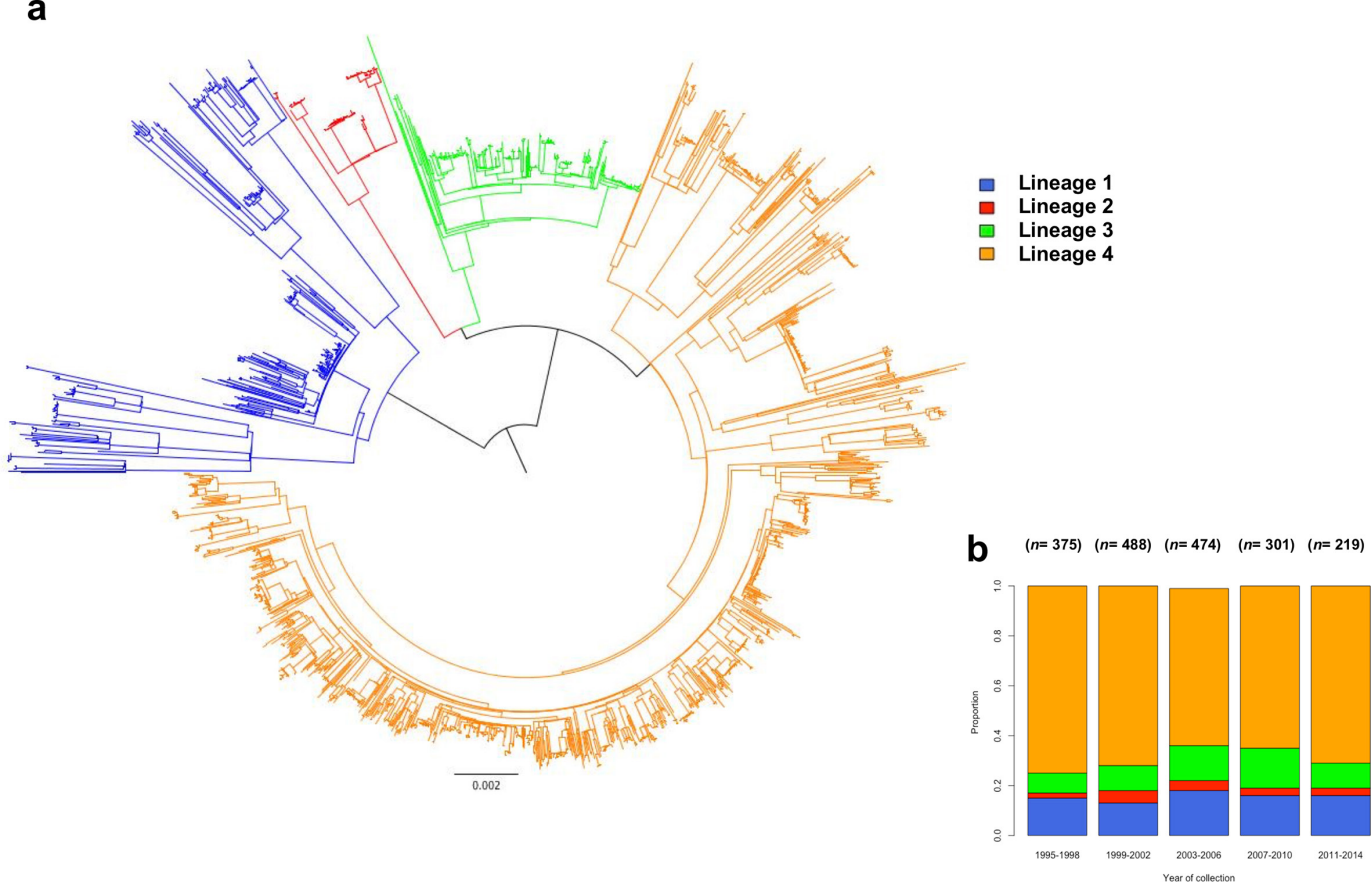
(a) Maximum-likelihood phylogenetic tree of the 1857 Karonga strains used in this study, showing lineages clustering into monophyletic groups. Lineage 1 is shown in blue, lineage 2 in red, lineage 3 in green and lineage 4 in orange. (b) The total number of sequenced cases and the proportion of strains collected, by lineage, over the study period 1995–2014 in 4-year categories.

**Fig. 2.**
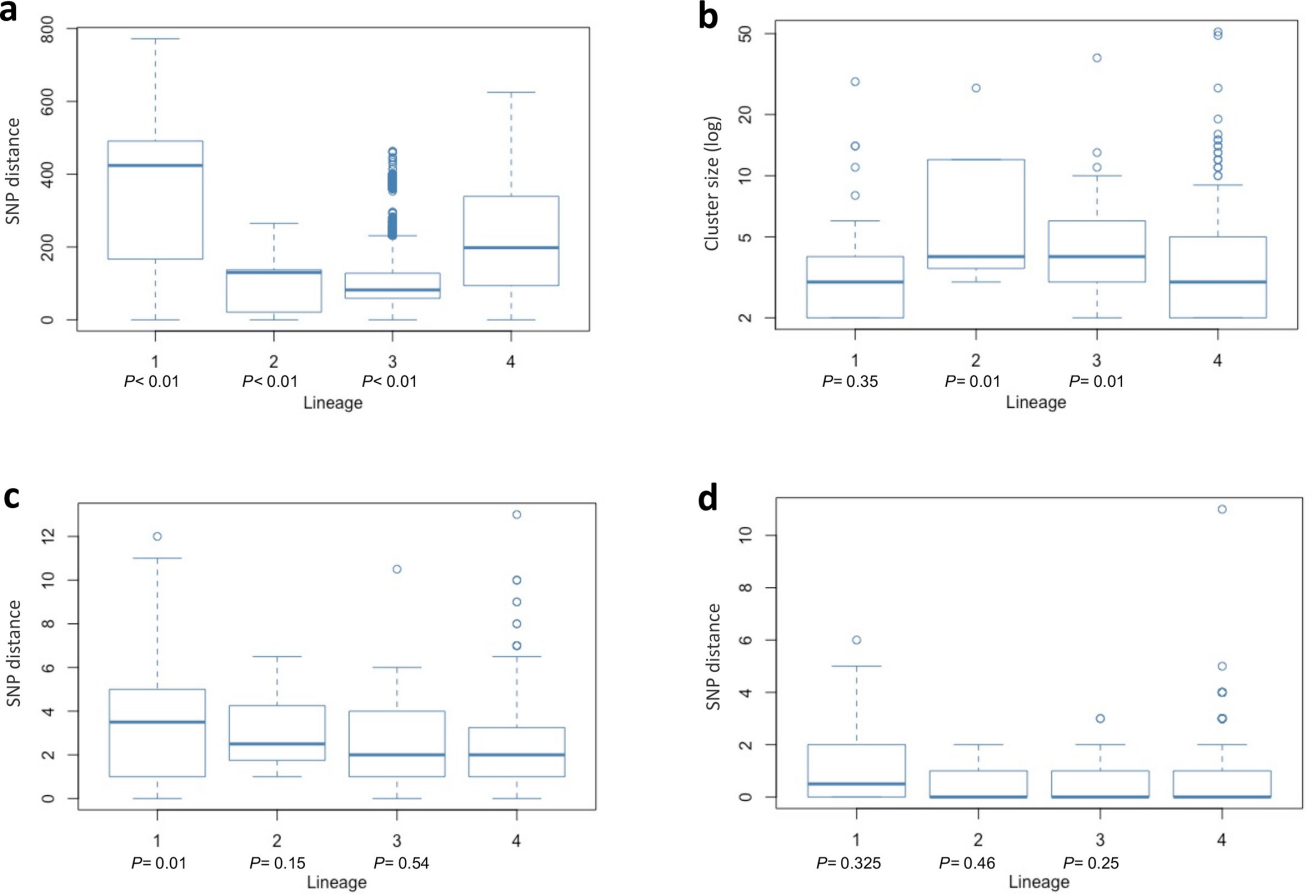
(a) Pairwise SNP distance by lineage between all Karonga strains used in the study. (b) Transmission cluster size by lineage, excluding non-clustered individuals. Clusters are defined as sampled strains (excluding non-sampled cases) linked either through direct transmission or with up to two transmission events inferred between individuals. *P*-values are a comparison against lineage 4 using the Wilcoxon rank sum test. (c) Median SNP distance in transmission clusters (sampled cases only). *P*-values are a comparison against lineage 4 using the Wilcoxon rank sum test. (d) Pairwise SNP distance between individuals in direct transmission events. *P*-values are a comparison against lineage 4 using the Wilcoxon rank sum test.

High-quality WGS data were available for 2129 *Mtb* genomes collected between 1995 and 2014. Multiple samples from the same individual during the same disease episode or through likely relapse [11] (*n*=77) or with a high likelihood of mixed infection (*n*=195) [12] were removed from further analysis, resulting in 1857 isolates being used in our analysis. The majority of isolates belonged to major lineage 4 (1279 isolates; 68.7 %), followed by lineages 1 (*n*=290; 15.6 %), 3 (*n*=217; 11.6 %) and 2 (*n*=71; 3.8 %) (Fig. 1a), with their proportions changing through the study period, but lineage 4 strains always being predominant (Fig. 1b). There were differences in diversity among strains of different major lineages, with lineages 2 and 3 being characterized by the least pairwise diversity [median=82 SNPs (interquartile range, IQR, 59–128) and 130 SNPs (IQR 21–137), respectively], followed by lineage 4 [median=198 SNPs (IQR 94–339)] and lineage 1 with the highest diversity between isolates [median=424 SNPs (IQR 167–491)] (Fig. 2a).

### Transmission networks

**Fig. 3.**
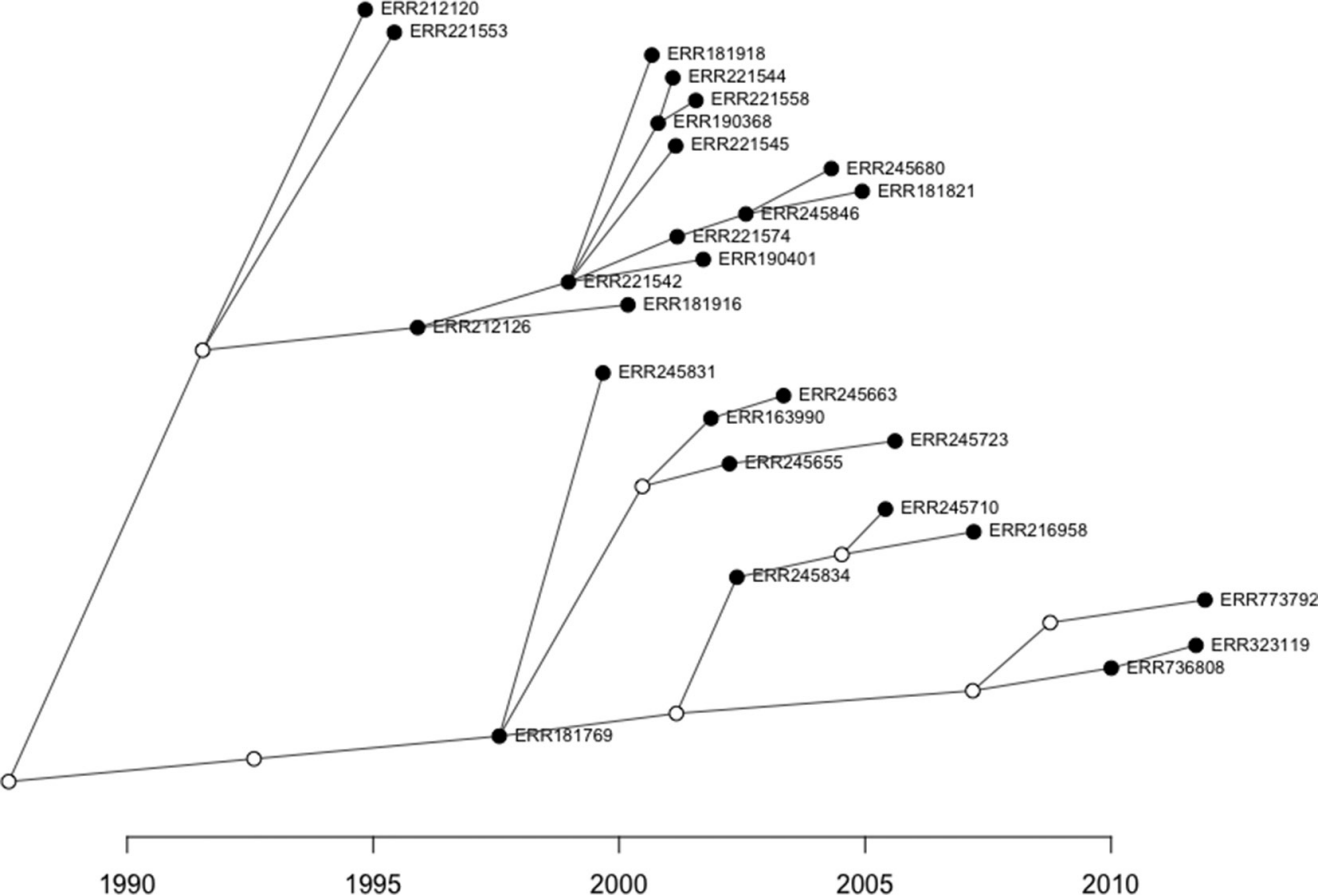
An example transmission network of lineage 2 strains reconstructed with TransPhylo software. Sampled cases are illustrated by filled dots and non-sampled, inferred intermediate hosts are indicated with unfilled dots. The direction of transmission is from left to right with the transmission date on the *x*-axis.

We found that 1355 isolates (73.0 %) were clustered into 281 transmission networks (or 57.8 % of isolates using the n-1 method removing the index case [33]) after removing likely relapse cases as previously described [11]. Fig. 3 shows an example of an inferred transmission network. Lineage 2 strains had the highest clustering rate (69/71 strains; 97.2 %), followed by lineage 3 (178/217 strains; 82.0 %), lineage 4 (911/1279; 71.2 %) and lineage 1 (197/290; 67.9 %). Cluster sizes ranged from 2 to 51 samples (median 3 cases, IQR 2–5) and were largest for lineage 2 strains (median 4 cases, IQR 4–12) (Fig. 2b). Strains belonging to lineage 1 were found in the smallest clusters (median 3 cases, IQR 2–4) with the highest genetic diversity (3.75 SNPs; IQR 1–5) (Fig. 2c). Lineage 4 had the lowest genetic diversity within clusters (median 2 SNPs, IQR 1–3.25), although this was not significantly different from lineage 2 and 3 clusters (Wilcoxon *P*=0.15 and 0.54, respectively) and was likely due to the significantly smaller average cluster size (Wilcoxon *P*<0.01).

We identified 544 direct transmission events between sampled cases (i.e. with no inferred unsampled host in-between), as detailed in the Supplementary Material S1. The time between direct transmission events ranged from 41 days to 12.4 years, with a median of 1.7 years (IQR 0.9–3.2 years). The median SNP difference between cases inferred to be linked by a direct transmission event was relatively low, with a median of 0 SNPs (IQR 0–1), and this was consistent between direct transmissions in different lineage clusters (Fig. 2d). The number of SNP differences between directly linked cases ranged from 0 to 12 SNPs, although the majority of cases differed by fewer than 5 SNPs (99.1 %). We also found no INDEL differences between directly linked cases.

### Recent infection

Recent infection was defined as transmission links for which the source case was within 3 years of the recipient, estimated by TransPhylo. Cases occurring before 1999 were excluded to ensure that the prior 3 years of data were available. We found that 49.4 % of cases showed evidence of recent infection (Table 1), with lineage 2 cases having the highest proportion (71.9 %) and lineage 1 (39.6 %) the lowest. We also found significant associations with age, with a higher proportion of recent infection in the younger age groups but no significant reduction in the proportion of recent infection over time.

**Table 1.** Demographic characteristics associated with recent infection, defined as cases for whom the source of infection has been inferred within the last 3 years. Cases prior to 1999 are excluded. Odds ratios and *P*-values are calculated through logistic regression and Wald chi-Squared test, adjusted for age, sex, year and lineage

	Recent infection/total	% recent infection	Odds ratio (95 % CI)	*P*-value
**Year**				
1999–2001	181/367	49.3	1	
2002–2004	171/379	45.1	0.9 (0.6–1.2)	
2005–2007	137/298	46.0	0.9 (0.7–1.2)	
2008–2010	93/219	42.5	0.8 (0.5–1.1)	
2011–2014	108/219	49.3	1.0 (0.7–1.4)	0.52
**Lineage**				
1	86/232	37.1	0.7 (0.5–0.9)	
2	46/64	71.9	2.8 (1.7–5.1)	
3	88/187	47.1	1.0 (0.7–1.4)	
4	470/999	47.0	1	<0.01
**Age group (years**)				
<20	33/64	51.6	1.8 (1.0–3.1)	
20–29	204/370	55.1	2.1 (1.5–2.9)	
30–39	236/528	44.7	1.4 (1.0–1.9)	
40–49	132/290	45.5	1.4 (1.0–2.0)	
50+	85/230	37.0	1	<0.01
**Sex**				
Female	361/752	48.0	1	
Male	329/730	45.1	0.9 (0.7–1.1)	0.33
**HIV status**				
Negative	235/514	45.7	1	
Positive on ART	64/123	52.0	1.3 (0.8–1.9)	
Positive no ART	274/584	46.9	1.1 (0.8–1.3)	0.51
**Previous TB**				
Yes	101/167	60.5	1.9 (1.4–2.6)	
No	589/1315	44.8	1	<0.01
**TB type**				
Smear positive	540/1144	47.2	1	
Smear negative	135/294	45.9	0.9 (0.7–1.2)	
Extrapulmonary	15/44	34.1	0.5 (0.3–1.0)	0.09
**Outcome**				
Completed	485/1023	47.4	1	
Died	144/286	50.3	1.1 (0.9–1.5)	
Lost/transferred	55/149	36.9	0.6 (0.4–0.9)	0.02
**Isoniazid Resistance**				
Resistant	59/99	59.6	1.8 (1.2–2.7)	
Sensitive	609/1313	46.4	1	<0.01
**Rifampicin resistance**				
Resistant	5/12	41.7	0.7 (0.2–2.2)	
Sensitive	664/1399	47.5	1	0.65
**Recent Residence**				
Karonga	571/1158	49.3	1	
Other Malawi	84/228	36.8	0.6 (0.4–0.8)	
Other country	21/66	31.8	0.5 (0.3–0.8)	<0.01
**Birthplace**				
Karonga	453/906	50.0	1	
Other Malawi	121/300	40.3	0.7 (0.5–0.9)	
Other country	102/252	40.5	0.7 (0.5–0.9)	<0.01

ART, antiretroviral therapy.

The risk of recent infection was higher in patients who had had a previous episode of TB [odds ratio adjusted for age, sex, year and lineage (aOR)=2.0 (95 % CI: 1.4–2.9; *P*<0.01)] compared to no previous episode. Recent infection was also lower in patients from outside Karonga district (though this may be due to sampling bias as the source of infection is less likely to be captured as it may be outside the sample population), and higher in patients with isoniazid-resistant *Mtb* [aOR=1.6 (95 % CI 1.0–2.5; *P*=0.01)]. There was also some evidence of a lower proportion of recent infection in extrapulmonary TB cases [aOR=0.5 (95 % CI 0.3–1.0; *P*=0.06] compared to smear-positive cases and in patients who were subsequently lost or transferred versus those completing treatment or who died.

By increasing the number of years from 3 to 5 to characterize recent infection, all associated factors identified with recent infection at 3 years were also found (Supplementary Material S2, Table S1). In addition, we found that HIV-positive patients were more likely to have recent infection than HIV-negative patients.

### Large clusters

**Fig. 4.**
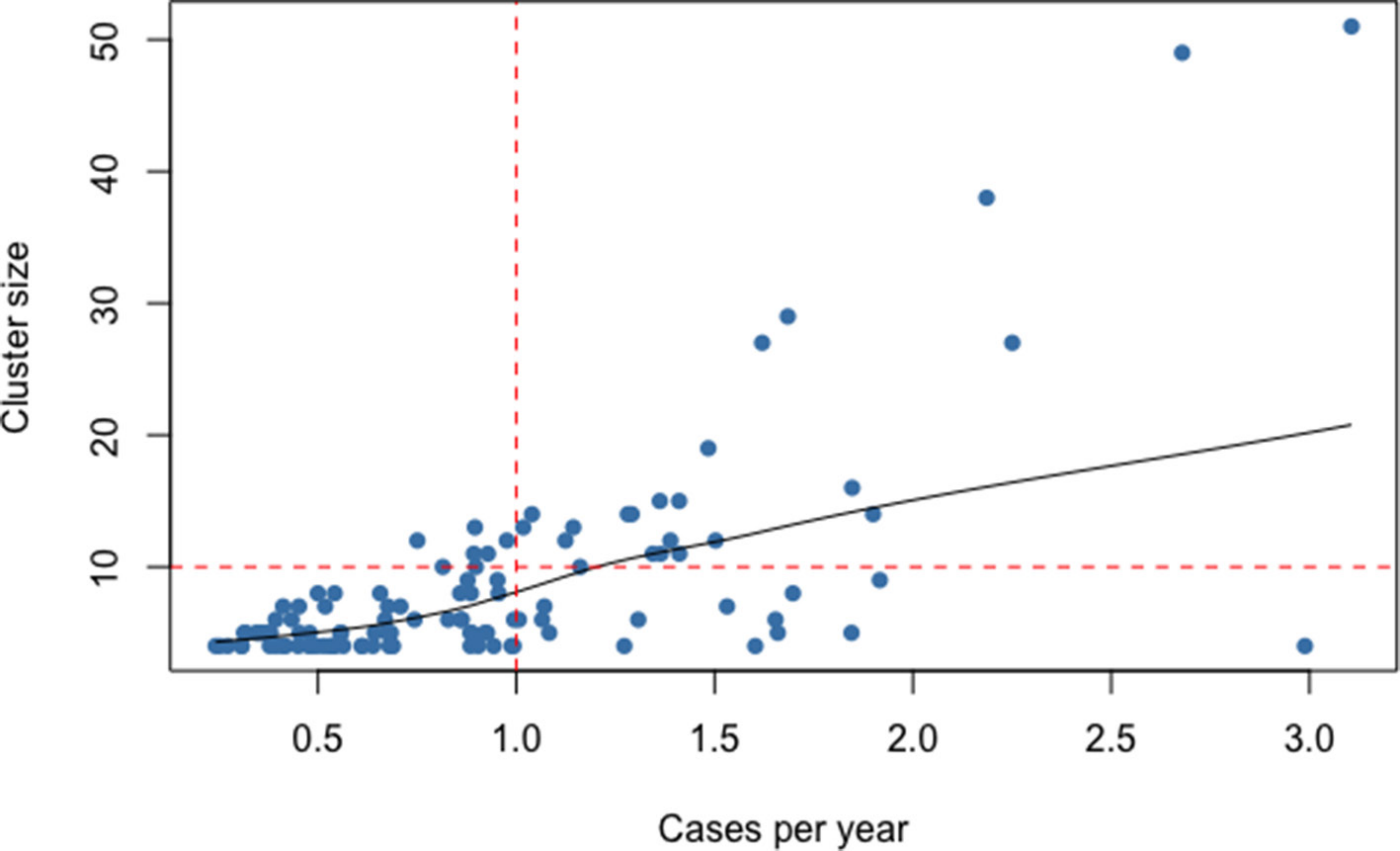
Cluster size by the number of cases per year (calculated as the date range of a cluster divided by the number of cases) in clusters with ≥five cases. Large clusters are defined as clusters of ≥10 cases and >1 case per year (red dashed lines), and are shown in the upper right quadrant of the figure.

Cases within large, persistent transmission clusters (>10 cases and a rate of >1 case per year, Fig. 4) were compared to small clusters (<5 cases) and unclustered cases. This resulted in 23 large clusters of between 10 and 51 cases. Intermediate-sized clusters (>10 cases but <1 case per year, or between 5 and 10 cases) were excluded from this analysis to allow for a more distinct comparison. Clusters with the earliest collection date after 2010 were also excluded as these may have the potential to continue growing into larger clusters after the study period.

Lineage 2 strains were significantly more likely to be found in large clusters [aOR=3.4 (95 % CI 1.9–6.3)], compared to lineage 4. The oldest patients (50+ years) were least likely to have large cluster strains. Residence within Karonga district was associated with large cluster strains, though again this is likely due to sampling bias, as cases within a cluster from outside the Karonga district would not be captured within our sample population. Additionally, there was some evidence that HIV-negative patients and those with rifampicin-resistant *Mtb* strains were less likely to be in large clusters (Table 2).

**Table 2.** Demographic characteristics associated with large clusters (cases in clusters of ≥10 cases and >1 case per year). Cases in clusters ≥10 cases but <1 case per year, and cases in clusters with the index case after 2010, are excluded. Odds ratios are calculated through logistic regression and *P*-values by the Wald chi-squared test, adjusted for age, sex, year and lineage

	Large clusters/total	% large clusters	Odds ratio (95 % CI)	*P*-value
**Year**				
1995–1998	81/304	26.6	1	
1999–2001	80/302	26.5	1.0 (0.7–1.4)	
2002–2004	95/303	31.4	1.2 (0.9–1.8)	
2005–2007	82/247	33.2	1.4 (0.9–2.0)	
2008–2010	57/184	31.0	1.2 (0.8–1.8)	
2011–2014	52/188	27.7	1.0 (0.7–1.5)	0.39
**Lineage**				
1	69/257	26.8	0.9 (0.7–1.3)	
2	27/47	57.4	3.4 (1.9–6.3)	
3	59/176	33.5	1.3 (0.9–1.8)	
4	292/1048	27.9	1	<0.01
**Age group (years**)				
<20	23/68	33.8	1.9 (1.0–3.4)	
20–29	130/403	32.6	1.8 (1.2–2.6)	
30–39	155/527	29.4	1.5 (1.1–2.2)	
40–49	89/299	29.8	1.5 (1.0–2.3)	
50+	50/231	21.6	1	0.05
**Sex**				
Female	220/761	28.9	1	
Male	227/767	29.6	1.0 (0.8–1.3)	0.80
**HIV status**				
Negative	131/503	26.0	1	
Positive on ART	40/108	37.0	1.6 (1.0–2.6)	
Positive no ART	187/583	32.1	1.3 (1.0–1.8)	0.08
**Previous TB**				
Yes	55/148	37.2	1.5 (1.0–2.1)	
No	392/1380	28.4	1	0.12
**TB type**				
Smear-positive	254/818	31.1	1	
Smear-negative	79/294	26.9	0.9 (0.6–1.1)	
Extrapulmonary	37/163	22.7	0.8 (0.4–1.3)	0.38
**Outcome**				
Completed	307/1003	30.6	1	
Died	96/315	30.5	1.0 (0.8–1.3)	
Lost/transferred	43/190	22.6	0.7 (0.5–1.0)	0.11
**Isoniazid resistance**				
Resistant	24/99	24.2	0.7 (1.0–1.2)	
Sensitive	408/1366	29.9	1	0.20
**Rifampicin resistance**				
Resistant	1/15	6.7	0.2 (0.0–0.9)	
Sensitive	432/1451	29.8	1	0.09
**Recent residence**				
Karonga	360/1114	32.3	1	
Other Malawi	51/248	20.6	0.5 (0.4–0.8)	
Other country	16/89	18.0	0.5 (0.3–0.8)	<0.01
**Birthplace**				
Karonga	281/907	31.0	1	
Other Malawi	85/315	27.0	0.8 (0.5–1.1)	
Other country	69/264	26.1	0.8 (0.6–1.1)	0.21

ART, antiretroviral therapy.

### Transmissibility

Infectors were defined as cases that were inferred to have transmitted *Mtb* to either sampled or unsampled cases. Demographic factors associated with these cases were compared to those with no onward transmission. Extrapulmonary cases, and those seen after 2010, which may transmit after the study period, were excluded. Four hundred and sixty-eight out of 1556 (30.1 %) cases seen before 2011 transmitted *Mtb* to at least 1 person; and 369 (23.7 %) transmitted with a serial interval of 4 years or less. Most infectors that transmitted within 4 years were responsible for a single onward transmission (80.2 %), with the number of secondary cases caused by infectors ranging from 1 to 6.

Table 3 shows the demographic associations with transmission to another case within 4 years, applying ordered logistic regression in relation to the number of secondary cases caused by each infector. There were lineage differences associated with transmissibility, with lineage 2 strains the most likely to transmit and those from lineage 1 the least likely. The proportion of infector cases in the total population decreased significantly per year throughout the duration of the study period (*P*<0.05) (Supplementary Material S2, Fig. S1). Patients under 50 years of age and those with smear-positive disease were more likely to transmit. Patients who died or were transferred/lost to follow-up, those with isoniazid resistance and those born outside Karonga District were less likely to transmit. Associations were similar when comparing all transmission events, not restricting to those giving rise to cases within 4 years (Supplementary Material S2, Table S2).

**Table 3.** Demographic characteristics associated with infector cases that have transmitted to another sampled or non-sampled case within 4 years. Cases collected after 2010 and extrapulmonary cases are excluded. Odds ratios are calculated through ordered logistic regression by number of secondary infections, and *P*-values by the Wald chi-squared test, adjusted for age, sex, year and lineage

	Infector /total	% infectors	Odds ratio (95 % CI)	*P*-value
**Year**				
1995–1998	99/337	29.4	1	
1999–2001	95/346	27.5	0.9 (0.6–1.2)	
2002–2004	89/367	24.3	0.8 (0.6–1.1)	
2005–2007	61/288	21.2	0.6 (0.4–0.9)	
2008–2010	25/218	11.5	0.3 (0.2–0.5)	<0.01
**Lineage**				
1	48/243	19.8	0.8 (0.6–1.2)	
2	20/61	32.8	1.6 (0.9–2.7)	
3	48/185	25.9	1.4 (0.9–1.8)	
4	253/1067	23.7	1	0.05
**Age group (years**)				
<20	13/55	23.6	1.6 (0.7–3.2)	
20–29	116/411	28.2	2.1 (1.4–3.2)	
30–39	132/547	24.1	1.8 (1.2–2.7)	
40–49	69/290	23.8	1.8 (1.2–2.8)	
50+	39/253	15.4	1	0.01
**Sex**				
Female	206/815	25.3	1	
Male	163/741	22.0	0.8 (0.7–1.1)	0.18
**HIV status**				
Negative	112/469	23.9	1	
Positive on ART	14/78	17.9	1.0 (0.5–1.9)	
Positive no ART	155/639	24.3	1.0 (0.7–1.3)	1.0
**Previous TB**				
Yes	35/171	20.4	0.9 (0.6–1.3)	
No	334/1385	24.1	1	0.43
**TB type**				
Smear-positive	329/1218	27.0	1	
Smear-negative	40/338	11.8	0.4 (0.2–0.5)	<0.01
**Outcome**				
Completed	262/1011	25.9	1	
Died	67/337	19.9	0.6 (0.5–0.9)	
Lost/transferred	40/191	20.9	0.7 (0.4–1.0)	<0.01
**Isoniazid Resistance**				
Resistant	19/108	17.6	0.6 (0.4–1.0)	
Sensitive	347/1402	24.8	1	0.07
**Rifampicin resistance**				
Resistant	2/15	13.3	0.5 (0.1–1.8)	
Sensitive	363/1495	24.3	1	0.36
**Recent residence**				
Karonga	263/1131	23.3	1	
Other Malawi	59/247	23.9	1.0 (0.7–1.3)	
Other country	20/90	22.2	0.8 (0.5–1.4)	0.78
**Birthplace**				
Karonga	242/942	25.7	1	
Other Malawi	52/299	17.4	0.6 (0.4–0.8)	
Other country	66/281	23.5	0.9 (0.6–1.2)	0.02

ART, antiretroviral therapy

### Genomic associations with transmission

We used two complementary approaches to search for genetic variants associated with transmission, convergence testing (phyC) and GWAS analysis. We considered both individual SNPs and whole-gene variation (through aggregated SNPs) to identify significant differences between variants found in transmitting versus non-transmitting strains. Non-transmitted strains were classified as singletons that were not included in any transmission cluster (*n*=409), again excluding extrapulmonary strains and cases collected after 2010. Transmitted strains were defined as strains from cases that transmitted to at least one other host within 4 years (*n*=369). SNPs identified in known antimicrobial resistance loci and *PE/PPE* genes, which were excluded in the transmission network analysis, were also considered to determine if these sites were associated with a transmissible phenotype. We also looked for convergent evolution in the presence or absence of small INDELs in the transmitter and non-transmitter groups.

The phyC analysis revealed no SNPs or whole genes that were convergently evolved in transmissible *Mtb* strains in our population. We also applied the analysis to lineage 1 and 4 strains separately (sample sizes in lineages 2 and 3 were too small to analyse) and no significant lineage-specific associations were identified. We found significant convergent evolution in a four-codon deletion in *PPE54* (*P*=0.01, Table 4)*,* a PE/PPE gene that has been proposed to be associated with antibiotic resistance through gene–gene interactions with other resistance loci [34], and in host–pathogen interactions [35].

**Table 4.** Genomic variants related to transmissibility identified through evolutionary convergence testing (phyC) and genome-wide association study (GWAS) mixed model approach (SNPs) and aggregated mutation mixed model (gene)

Locus tag	Gene name	Function	*P*-value	Identification method	SNP, gene, insertion or deletion
Rv3343c	*PPE54*	*PE*/*PPE* gene	0.01	phyC	Δ12 bp deletion
Rv3751	.	Probable integrase	8.8×10^−5^	GWAS	S* SNP (A31A)
Rv0974c	*accD2*	Acetyl-CoA carboxylase	2.6×10^−4^	GWAS	NS** SNP (N125H)
Rv3812	*PE_PGRS62*	*PE*/*PPE* gene	3.1×10^−4^	GWAS	Gene
Rv0056	*rplI*	50S ribosomal protein L9	3.9×10^−4^	GWAS	Δ3 bp insertion
Rv2077c	.	Possible transmembrane protein	6.2×10^−4^	GWAS	Δ3 bp deletion

*, synonymous; **, non-synonymous.

A GWAS analysis revealed variants that are potentially linked to transmissibility (Table 4). The most statistically significant variant was the synonymous SNP A31A in *Rv3751,* a non-essential gene coding for a probable phage integrase (*P*=8.8×10^−5^). We found two further loci that were found to be less significantly associated with transmissibility in our data (*P*<10^−4^), a non-synonymous mutation N125H in *accD2* encoding an acetyl-CoA carboxylase subunit and the *PE*/*PPE* gene *Rv3812* identified through the gene-level, aggregated-mutation GWAS. In addition, we found fewer significant associations (*P*<10^−4^) in a 3 bp deletion in Rv2077c, a possible transmembrane protein, and a 3 bp insertion in *rplI*, which codes for the 50 s ribosomal protein L9. Five genomic regions previously identified as being involved in transmission in a low-burden setting [36] were not validated in our study (*P*>0.05; Supplementary Material S2, Table S3).

## Discussion

Accurate reconstruction and analysis of TB transmission networks can provide insights into the factors that can influence the spread of the infection. Here we present a comprehensive large-scale study of transmission in a high-incidence region. We applied a sophisticated two-step Bayesian approach to reconstruct TB transmission networks from WGS data, allowing for within-host evolution and non-sampled hosts between sampled cases [3], the absence of which has been a limitation in previous studies.

The characteristics of the resulting transmission networks are consistent with a population with some latent infection, with a median generation time between transmission events of 1.7 years (IQR 0.9–3.2 years). The median distance between directly linked cases ranged from 0 to 12 SNPs, supporting a previous estimate found in this population between index patients and their identified prior contacts [8]. Network reconstruction has allowed us to gain further insights into the demographic factors associated with transmission in this setting and identify genomic variants that may be associated with transmissibility.

There are limitations with the methods used to infer transmission events and identify infector strains. While intermediate hosts between sampled cases were inferred, transmission from a sampled case to a non-sampled case that does not transmit onwards would not be captured and, in this scenario, the sampled case will not be characterized as an infector. Furthermore, our measure of transmission is necessarily time-limited: disease may occur up to a lifetime after infection, whereas we have taken a 4-year window, so underestimating transmission. However, the approach used in this study is a huge step towards a more complete reconstruction of transmission to reveal associated risk factors, including in cohorts with incomplete sampling, which can be used to prioritize patients and their contacts with a high possibility of onward transmission.

We found clear lineage differences in transmissibility and recent infection in our population, with lineage 2 strains exhibiting the greatest proportion of recently infected cases and the highest transmissibility, followed by lineage 3 strains. These lineages were also characterized by larger transmission clusters of cases with low genetic diversity. Increased transmission of lineage 2 strains, particularly in the Beijing subtype, has been reported in previous work in the Karonga population [6], as well as studies in other regions [37–39]. 6 In this study though, we do not see evidence that lineage 2 strains increase in frequency, with a peak frequency early in study period around 1999–2002. This may be driven by high local transmission in a small number of lineage 2 clusters with few imported cases that are persisting in the study area, in contrast to lineage 4 strains that are more dominant in the region and account for the majority of imported cases from outside Karonga [6], and thus will include a greater number of unlinked cases. Lineage 1 strains were found in small clusters composed of significantly more diverse strains and were the least transmissible, with the lowest proportion of recent infection. This supports evidence that lineage 1 strains, which are considered ‘ancient’, have lower virulence than the ‘modern’ lineages 2, 3 and 4 [40].

The decrease in transmission by calendar year reported previously up to the year 2010 [6] is confirmed in this new analysis and has continued from 2011 to 2014, though surprisingly it is not seen in the proportion of disease due to recent infection in the latest period. The lowest proportion of recent infection and transmission was found in those aged more than 50 years and this may reflect an increase in relapse shown previously [11], or the higher risk of reactivation due to a weakened immune system with age [41], or the lower chance of catching or spreading the infection as mobility and contact outside the home decreases.

We also looked at the underlying factors that characterize larger, persistent transmission clusters that have been circulating in the population over a long period, defined as clusters with >10 cases transmitting at a rate of >1 case per year. The associations with age, lineage and residence were similar to those found in other analyses [42]. Interestingly, HIV-positive patients were more likely to have large cluster strains, whereas the evidence of association between HIV infection and recent infection was weaker. It is possible that nosocomial transmission in HIV testing and treatment centres has contributed to these clusters [43]. Although it has been suggested that HIV-positive patients transmit less than HIV-negative patients, because they tend to be diagnosed earlier or die quickly, we found no evidence of reduced transmission.

Comparing the full genomic variation in strains that have been identified as transmitting to at least one recipient against cases with no evidence of onward transmission, we were able to look for possible mutations that were positively associated with transmissible strains. Two complementary methods, phyC and GWAS, were employed to test for differences between these groups, both in single-site mutations (SNPs and INDELS) and in aggregated mutation differences within whole genes. The GWAS approach revealed SNPs in *Rv3751*, *accD2*, *Rv3812*, *rplI* and Rv2077c as potentially associated with transmissibility. Members of the *accD* gene family have been linked to persistence of the *Mtb* bacterium [44]. The *PE/PPE* gene *Rv3812* has a role in the host immune response and virulence of the pathogen [45]. The phyC evolutionary convergence approach revealed a 12 bp deletion within *PPE54*, validated through manual inspection of alignment files and *de novo* assemblies of isolates, which is an important gene for *in vitro* growth and host–pathogen interactions [35], as well as isoniazid and rifampicin resistance [34].

Previous work using a *phyC* approach identified five genomic regions involved in transmission in a low-burden setting in the Netherlands. None were validated in our analysis, although we did have poor sequencing coverage in *Rv2815–2816* c and *Rv3512*, so we can not explicitly rule out a role in transmissibility within our population (Supplementary Material S2, Table S3). These differences may be explained by disease burden (low-burden vs high-burden setting), methodologies (the transmissibility phenotype in the Netherlands population was defined using a combination of clustering through DNA fingerprinting and a measure of transmission risk using demographic features) and genetic variability within the two sample populations [36].

In general, any loci associated with transmissibility need to be validated in other cohorts and settings, and experimentally. Our dataset contained few lineage 2 and 3 strains that did not cluster in transmission networks, limiting our ability to conduct lineage-specific association tests. The higher transmission found in these lineages may be driven by lineage-specific genomic variation that may be revealed by testing cohorts with a high proportion of lineage 2 and 3 strains, including those sourced from East Asia and the Indian subcontinent. Putative variants found to be associated with transmissibility through genomic analysis can be experimentally validated using the CRISPR/cas9 system, with methods specific to gene modification in *Mycobacterium* species beginning to be developed [46].

There is a body of evidence to suggest that successful transmission and virulence in *Mtb* can be affected by mutation in key resistance-conferring genes, with both higher and lower levels of transmission depending on the setting and strain resistance profiles [47–50]. In this analysis, isoniazid-resistant strains were less likely to transmit but more likely to be due to recent transmission, but there were few rifampicin-resistant strains. It is also likely that in a high-burden setting host demographic and genetic factors will play a larger role in disease susceptibility, the spread of infection and the development of disease as individuals will be exposed to higher levels of contact with potentially infectious individuals [51, 52]. As such, the likelihood of transmission from host to recipient is not isolated to the pathogenic virulence and fitness but also to host–pathogen interactions, and this may go towards explaining the small number of *Mtb* genes found to be associated with transmission in our population. Incorporating host genome differences and interactions with pathogen in work of this nature would offer a more complete understanding of the underlying factors influencing the spread of *Mtb*.

## Conclusions

Our work applied a sophisticated Bayesian approach to reconstruct TB transmission networks with high confidence in high-burden settings, using WGS data from a large-scale, long-term study. It has built upon a previous study detailing transmission in the region [6] by extending the study for a longer period and updating the methods for transmission reconstruction using recent advances. We have validated previous findings to support increased transmissibility of lineage 2 and 3 strains. In addition, we found evidence that differences in age and lineage can influence the likelihood of large, persistent transmission clusters. We identified three potential genes that may be associated with transmissible strains of *Mtb* based on significant associations with transmissibility in our population and previous studies on their function [34, 35, 44, 45], which can be used as potential target sites for further validation studies. While this work highlights the complex nature of identifying the drivers of transmission in TB populations, the methods applied can be used as a framework for future studies, where ideally both host and pathogen genomic components should be integrated. Ultimately, any insights gained into genetic and non-genetic factors linked to transmissibility have the potential to inform much-needed disease control and management strategies to reduce the spread of TB globally.

## Data Bibliography

1. Karonga *Mycobacterium tuberculosis* samples: European Nucleotide Archive (ENA) short-read archive (project ID ERP000436 and ERP001072).

2. *M. tuberculosis* reference strain H37Rv GenBank accession number NC_000962.3.

## Supplementary Data

Supplementary material 1

Supplementary material 2

## References

[1] WHO (2016). Global tuberculosis report. global tuberculosis report 2016.

[2] Eldholm V, Rieux A, Monteserin J, Lopez JM, Palmero D (2016). Impact of HIV co-infection on the evolution and transmission of multidrug-resistant tuberculosis. eLife.

[3] Didelot X, Fraser C, Gardy J, Colijn C, Malik H (2017). Genomic infectious disease epidemiology in partially sampled and ongoing outbreaks. Mol Biol Evol.

[4] Luo T, Yang C, Peng Y, Lu L, Sun G (2014). Whole-Genome sequencing to detect recent transmission of *Mycobacterium tuberculosis* in settings with a high burden of tuberculosis. Tuberculosis.

[5] Casali N, Nikolayevskyy V, Balabanova Y, Harris SR, Ignatyeva O (2014). Evolution and transmission of drug-resistant tuberculosis in a Russian population. Nat Genet.

[6] Guerra-Assunção JA, Fine PEM, Crampin AC, Houben R, Mzembe T (2015). Large-Scale whole genome sequencing of M. tuberculosis provides insights into transmission in a high prevalence area. Elife.

[7] Campos LC, Rocha MVV, Willers DMC, Silva DR, Vieira Rocha MV (2016). Characteristics of patients with smear-negative pulmonary tuberculosis (TB) in a region with high TB and HIV prevalence. PLoS One.

[8] Glynn JR, Guerra-Assunção JA, Houben RMGJ, Sichali L, Mzembe T (2015). Whole genome sequencing shows a low proportion of tuberculosis disease is attributable to known close contacts in rural Malawi. PLoS One.

[9] Mboma SM, Houben RMGJ, Glynn JR, Sichali L, Drobniewski F (2013). Control of (Multi)drug resistance and tuberculosis incidence over 23 Years in the context of a well-supported tuberculosis programme in rural Malawi. PLoS One.

[10] Crampin AC, Glynn JR, Fine PEM (2009). What has Karonga taught us? tuberculosis studied over three decades. Int J Tuberc Lung Dis.

[11] Guerra-Assunção JA, Houben RMGJ, Crampin AC, Mzembe T, Mallard K (2015). Recurrence due to relapse or reinfection With *Mycobacterium tuberculosis* : A whole-genome sequencing approach in a large, population-based cohort with a high HIV infection prevalence and active follow-up. J Infect Dis..

[12] Sobkowiak B, Glynn JR, Houben RMGJ, Mallard K, Phelan JE (2018). Identifying mixed *Mycobacterium tuberculosis* infections from whole genome sequence data. BMC Genomics.

[13] Jombart T, Eggo RM, Dodd PJ, Balloux F (2011). Reconstructing disease outbreaks from genetic data: a graph approach. Heredity.

[14] Didelot X, Gardy J, Colijn C (2014). Bayesian inference of infectious disease transmission from whole-genome sequence data. Mol Biol Evol.

[15] Walker TM, Ip CLC, Harrell RH, Evans JT, Kapatai G (2013). Whole-Genome sequencing to delineate Mycobacterium tuberculosis outbreaks: a retrospective observational study. Lancet Infect Dis.

[16] Lee RS, Radomski N, Proulx J-F, Manry J, McIntosh F (2015). Reemergence and amplification of tuberculosis in the Canadian Arctic. J Infect Dis..

[17] Yang C, Luo T, Shen X, Wu J, Gan M (2017). Transmission of multidrug-resistant *Mycobacterium tuberculosis* in Shanghai, China: a retrospective observational study using whole-genome sequencing and epidemiological investigation. Lancet Infect Dis.

[18] Roetzer A, Diel R, Kohl TA, Rückert C, Nübel U (2013). Whole genome sequencing versus traditional genotyping for investigation of a Mycobacterium tuberculosis outbreak: a longitudinal molecular epidemiological study. PLoS Med.

[19] Li H, Durbin R (2009). Fast and accurate short read alignment with Burrows-Wheeler transform. Bioinformatics.

[20] Li H, Handsaker B, Wysoker A, Fennell T, Ruan J (2009). The sequence Alignment/Map format and SAMtools. Bioinformatics.

[21] Phelan JE, Coll F, Bergval I, Anthony RM, Warren R (2016). Recombination in pe/ppe genes contributes to genetic variation in *Mycobacterium tuberculosis* lineages. BMC Genomics.

[22] Zerbino DR, Bateman A, Pearson WR, Stein LD, Stormo GD, Yates JR (2010). Using the velvet *de novo* assembler for short-read sequencing technologies. Current Protocols in Bioinformatics [Internet].

[23] Zerbino DR, Birney E (2008). Velvet: algorithms for *de novo* short read assembly using de Bruijn graphs. Genome Res.

[24] Seemann T (2014). Prokka: rapid prokaryotic genome annotation. Bioinformatics.

[25] Page AJ, Cummins CA, Hunt M, Wong VK, Reuter S, Reu- S (2015). Roary: rapid large-scale prokaryote pan genome analysis. Bioinformatics.

[26] Jajou R, de Neeling A, van Hunen R, de Vries G, Schimmel H (2018). Epidemiological links between tuberculosis cases identified twice as efficiently by whole genome sequencing than conventional molecular typing: a population-based study. PLoS One.

[27] Lieberman TD, Wilson D, Misra R, Xiong LL, Moodley P (2016). Genomic diversity in autopsy samples reveals within-host dissemination of HIV-associated *Mycobacterium tuberculosis*. Nat Med.

[28] Drummond AJ, Suchard MA, Xie D, Rambaut A, Bouckaert RR, Dong X (2012). Bayesian phylogenetics with BEAUti and the beast 1.7. Mol Biol Evol.

[29] Stamatakis A (2014). RAxML version 8: a tool for phylogenetic analysis and post-analysis of large phylogenies. Bioinformatics.

[30] Glynn JR, Vyonycky E, Fine PEM (1999). Influence of sampling on estimates of clustering and recent transmission of *Mycobacterium tuberculosis* derived from DNA fingerprinting techniques. Am J Epidemiol.

[31] Farhat MR, Shapiro BJ, Kieser KJ, Sultana R, Jacobson KR (2013). Genomic analysis identifies targets of convergent positive selection in drug-resistant *Mycobacterium tuberculosis*. Nat Genet.

[32] Coll F, Phelan J, Hill-Cawthorne GA, Nair MB, Mallard K (2018). Genome-Wide analysis of multi- and extensively drug-resistant Mycobacterium tuberculosis. Nat Genet.

[33] Van SC, Cule M, Welte A, Van HP, Der SGV (2012). Towards eliminating bias in cluster analysis of TB genotyped data. PLoS One.

[34] Cui Z-J, Yang Q-Y, Zhang H-Y, Zhu Q, Zhang Q-Y (2016). Bioinformatics identification of drug resistance-associated gene pairs in *Mycobacterium tuberculosis*. Int J Mol Sci.

[35] Chiliza TE, Pillay M, Pillay B (2017). Identification of unique essential proteins from a *Mycobacterium tuberculosis* F15 / Lam4 / KZN phage secretome library. Pathog Dis.

[36] Nebenzahl-Guimaraes H, van Laarhoven A, Farhat MR, Koeken VACM, Mandemakers JJ (2017). Transmissible *Mycobacterium tuberculosis* strains share genetic markers and immune phenotypes. Am J Respir Crit Care Med.

[37] Parwati I, van Crevel R, van Soolingen D, Van CR, Van SD (2010). Possible underlying mechanisms for successful emergence of the *Mycobacterium tuberculosis* Beijing genotype strains. Lancet Infect Dis.

[38] Niemann S, Diel R, Khechinashvili G, Gegia M, Mdivani N (2010). *Mycobacterium tuberculosis* Beijing lineage favors the spread of multidrug-resistant tuberculosis in the Republic of Georgia. J Clin Microbiol.

[39] Holt KE, McAdam P, Thai PVK, Thuong NTT, Ha DTM (2018). Frequent transmission of the *Mycobacterium tuberculosis* Beijing lineage and positive selection for the EsxW Beijing variant in Vietnam. Nat Genet.

[40] Reiling N, Homolka S, Walter K, Brandenburg J, Niwinski L (2013). Clade-Specific virulence patterns of *Mycobacterium tuberculosis* complex strains in human primary macrophages and aerogenically infected mice. MBio.

[41] Byng-Maddick R, Noursadeghi M (2016). Does tuberculosis threaten our ageing populations?. BMC Infect Dis.

[42] Glynn JR, Crampin AC, Traore H, Chaguluka S, Mwafulirwa DT, Drobniewski FD (2008). Determinants of cluster size in large, population-based molecular epidemiology study of tuberculosis, Northern Malawi. Emerg Infect Dis.

[43] Mzembe T, Mclean E, Khan PY, Koole O, Sichali L (2018). Risk of *Mycobacterium tuberculosis* transmission in an antiretroviral therapy clinic. AIDS.

[44] Gande R, Gibson KJC, Brown AK, Krumbach K, Dover LG (2004). Acyl-CoA Carboxylases (*accD2* and *accD3*), together with a unique polyketide synthase (*Cg-pks*), are key to mycolic acid biosynthesis in *Corynebacterianeae* such as *Corynebacterium glutamicum* and *Mycobacterium tuberculosis*. J Biol Chem.

[45] Vani J, Shaila MS, Trinath J, Balaji KN, Kaveri S (2013). *Mycobacterium tuberculosis* cell Wall–Associated Rv3812 protein induces strong dendritic Cell–Mediated interferon γ responses and exhibits vaccine potential.. J Infect Dis.

[46] Rock JM, Hopkins FF, Chavez A, Diallo M, Chase MR, Michael R (2017). Programmable transcriptional repression in mycobacteria using an orthogonal CRISPR interference platform. Nat Microbiol.

[47] Gagneux S (2009). Fitness cost of drug resistance in *Mycobacterium tuberculosis*. Clin Microbiol Infect.

[48] Gagneux S, Burgos MV, DeRiemer K, Enciso A, Muñoz S (2006). Impact of bacterial genetics on the transmission of isoniazid-resistant *Mycobacterium tuberculosis*. PLoS Pathog.

[49] Hu Y, Hoffner S, Jiang W, Wang W, Xu B (2010). Extensive transmission of isoniazid resistant *M. tuberculosis* and its association with increased multidrug-resistant TB in two rural counties of eastern China: a molecular epidemiological study. BMC Infect Dis.

[50] Nieto R LM, Mehaffy C, Creissen E, Troudt J, Troy A, Bielefeldt-ohmann H (2016). Virulence of *Mycobacterium* tuberculosis after acquisition of isoniazid resistance: individual nature of katG mutants and the possible role of AhpC. PLoS One.

[51] Gagneux S (2012). Host–pathogen coevolution in human tuberculosis. Phil. Trans. R. Soc. B.

[52] Palittapongarnpim P, Ajawatanawong P, Viratyosin W, Smittipat N, Disratthakit A (2018). Evidence for host-bacterial co-evolution via genome sequence analysis of 480 Thai *Mycobacterium tuberculosis* lineage 1 isolates. Sci Rep.

