# Supplementary material for: Bayesian reconstruction of Mycobacterium tuberculosis transmission networks in a high incidence area over two decades in Malawi reveals associated risk factors and genomic variants
Source: Microb Genom. 2020 Apr 1;6(4):e000361. doi: 10.1099/mgen.0.000361 (PMC7276699; doi:10.1099/mgen.0.000361)
Supplement: Supplementary material 2 [file mgen-6-361-s002.pdf]

SUPPLEMENTARY TABLES AND FIGURES

	Recent infection / Total	% Recent infection	Odds ratio (95% CI)	P–value
**Year**				
2001-2004	259 / 481	53.8	1	
2005-2007	156 / 298	52.3	1.0 (0.9 - 1.1)	
2008-2010	108 / 219	49.3	1.0 (0.9 - 1.0)	
2011-2014	124 / 219	56.6	1.0 (0.9 - 1.1)	0.49
**Lineage**				
1	85 / 202	42.1	0.6 (0.5 - 0.9)	
2	46 / 54	85.2	5.0 (2.5 - 11.7)	
3	87 / 159	54.7	1.1 (0.7 - 1.5)	
4	429 / 810	53.0	1	<0.01
**Age group (years)**				
< 20	28 / 52	53.8	1.6 (0.8 - 3.0)	
20-29	175 / 297	58.9	1.8 (1.2 - 2.6)	
30-39	227 / 439	51.7	1.3 (0.9 - 1.9)	
40-49	132 / 243	54.3	1.5 (1.0 - 2.2)	
50+	85 / 194	43.8	1	0.04
**Sex**				
Female	323 / 614	52.6	1.0 (0.8 - 1.3)	
Male	324 / 611	53.0	1	0.98
**HIV status**				
Negative	227 / 447	50.8	1	
Positive on ART	74 / 123	60.2	1.5 (1.0 - 2.2)	
Positive no ART	261 / 470	55.5	1.2 (0.9 - 1.6)	0.11
**Previous TB**				
Yes	90 / 143	62.9	1.6 (1.1 - 2.3)	
No	557 / 1074	51.9	1	0.01
**TB type**				
Smear positive	514 / 953	53.9	1	
Smear negative	124 / 239	51.9	0.9 (0.7 - 1.2)	
Extrapulmonary	9 / 25	36.0	0.5 (0.2 - 1.0)	0.17
**Outcome**				
Completed	469 / 868	54.0	1	
Died	126 / 219	57.5	1.2 (0.9 - 1.6)	
Lost/transferred	44 / 107	41.1	0.6 (0.4 - 0.9)	0.02
**Isoniazid Resistance**				
Resistant	51 / 81	63.0	1.5 (0.9 - 2.4)	
Sensitive	569 / 1066	53.4	1	0.09
**Rifampicin resistance**				
Resistant	6 / 11	54.5	1.0 (0.3 - 3.4)	
Sensitive	615 / 1135	54.2	1	0.97
**Recent Residence**				
Karonga	547 / 981	55.8	1	
Other Malawi	70 / 165	42.4	0.6 (0.4 - 0.8)	
Other Country	17 / 41	41.5	0.5 (0.3 - 1.0)	<0.01
**Birthplace**				
Karonga	418 / 756	55.3	1	
Other Malawi	114 / 237	48.1	0.8 (0.6 - 1.0)	
Other Country	101 / 203	49.8	0.8 (0.6 - 1.1)	0.09

Table S1. Demographic characteristics associated with recent infection, defined as cases for whom the source of infection has been inferred within the last 5 years. Cases prior to 2001 are excluded. Odds ratios and *p* values are calculated through logistic regression and Wald Chi-Squared test, adjusted for age, sex, year and lineage; ART = Antiretroviral Therapy

	Infector / Total	% Infectors	Odds ratio (95% CI) *	P–value *
**Year**				
1995-1998	135 / 337	40.0	1	
1999-2001	124 / 346	35.8	0.8 (0.6 - 1.1)	
2002-2004	106 / 367	28.9	0.6 (0.5 - 0.8)	
2005-2007	75 / 288	26.0	0.5 (0.4 - 0.7)	
2008-2010	28 / 218	12.8	0.2 (0.1 - 0.3)	< 0.01
**Lineage**				
1	68 / 243	28.0	1.0 (0.7 - 1.4)	
2	23 / 61	37.7	1.5 (0.9 - 2.6)	
3	67 / 185	36.2	1.6 (1.2 - 2.3)	
4	310 / 1067	29.0	1	0.022
**Age group (years)**				
< 20	15 / 55	27.3	1.4 (0.7 - 2.6)	
20-29	144 / 411	35.0	2.1 (1.5 - 3.1)	
30-39	167 / 547	30.5	1.8 (1.3 - 2.7)	
40-49	91 / 290	31.4	1.9 (1.3 - 2.9)	
50+	51 / 253	20.2	1	<0.01
**Sex**				
Female	260 / 815	31.9	1	
Male	208 / 741	28.1	0.8 (0.7 - 1.1)	0.13
**HIV status**				
Negative	135 / 469	28.8	1	
Positive on ART	17 / 78	21.8	1.2 (0.6 - 2.1)	
Positive no ART	202 / 639	31.6	1.1 (0.8 - 1.4)	0.74
**Previous TB**				
Yes	47 / 171	27.5	0.9 (0.7 - 1.3)	
No	421 / 1385	30.4	1	0.75
**TB type**				
Smear positive	421 / 1218	34.6	1	
Smear negative	47 / 338	13.9	0.3 (0.2 - 0.4)	< 0.01
**Outcome**				
Completed	324 / 1011	32.0	1	
Died	95 / 337	28.2	0.7 (0.5 - 0.9)	
Lost/transferred	49 / 191	25.7	0.6 (0.4 - 0.9)	< 0.01
**Isoniazid Resistance**				
Resistant	28 / 108	25.9	0.7 (0.5 - 1.1)	
Sensitive	437 / 1402	31.2	1	0.19
**Rifampicin resistance**				
Resistant	3 / 15	20.0	0.6 (0.1 - 1.9)	
Sensitive	461 / 1495	30.8	1	0.43
**Recent Residence**				
Karonga	325 / 1131	28.7	1	
Other Malawi	81 / 247	32.8	1.1 (0.8 – 1.5)	
Other Country	26 / 90	28.9	0.8 (0.5 – 1.4)	0.67
**Birthplace**				
Karonga	304 / 942	32.3	1	
Other Malawi	70 / 299	23.4	0.6 (0.5 – 0.9)	
Other Country	81 / 281	28.8	0.8 (0.6 – 1.1)	0.02

Table S2. Demographic characteristics associated with infector cases, classified as transmitting (to any number of secondary cases) or not transmitting. Cases after 2010 and extrapulmonary cases are excluded. Odds ratios and *p* values are calculated through logistic regression and Wald Chi-Squared test, adjusted for age, sex, year and lineage; ART = Antiretroviral Therapy

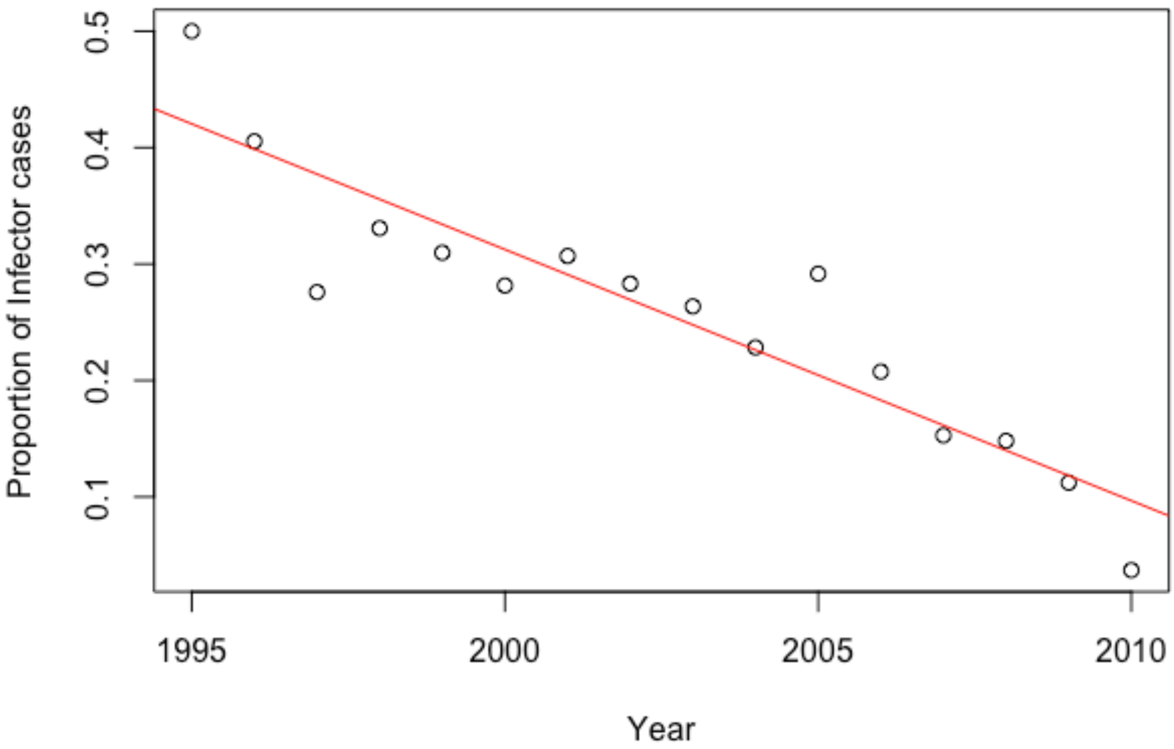

Figure S1. Trend showing the significant decrease in the proportion of transmitter strains (cases transmitting to another case within 4 years) per total cases per year between 1995 – 2010. Linear regression adjusted R squared = 0.82, p < 0.01.

Gene name	Variant in non-transmitters (n = 409)	Variant in transmitters (n = 369)	Percentage of missing calls
Rv0197			
232238 G>A	3	2	0.5
232361 A>G	6	0	0.1
232680 C>A	2	0	0.1
232851 G>A	1	1	0.1
232978 G>A	3	2	0.1
233083 G>A	1	2	0.1
233196 G>A	0	2	0.1
233751 A>G	4	1	0.0
233942 C>T	8	11	0.0
234029 T>G	4	3	0.0
234167 C>A	2	0	0.0
234477 T>G	*406*	*369*	0.3
234508 G>A	4	0	0.0
Rv2813–2814c			
3119137 T>G	0	3	0.1
3119720 G>C	0	3	1.7
Rv2815–2816c			
3122005 A>C	4	1	70.9
3123242 C>A	6	1	26.1
3123422 A>T	3	2	6.7
PE-PGRS56 (Rv3512)			
3941967 G>A	2	1	20.8
3942238 G>A	6	3	28.9
3942655 G>A	8	11	2.6
3942875 G>C	2	1	34.7
3943019 C>G	269	212	35.0
3943601 C>T	2	0	63.4
3944287 C>A	2	1	37.5
3944846 G>A	1	4	23.1
3943126 Δ 18bp	11	1	53.9
3943259 Δ 9bp	0	4	38.1
3943649 Δ 3bp	2	0	57.5
3943744 Δ 9bp	14	1	73.8
3944342 Δ 9bp	1	1	42.0
3944465 Δ 1bp	13	0	45.3
espE (Rv3864)			
4340286 G>A	8	11	0.1
4340312 A>G	19	2	0.1
4340330 T>G	*227*	*181*	0.1
4340519 T>C	0	3	0.1

4340992 G>A	2	0	0.3
4341029 C>T	0	4	0.1
4341396 + 12bp	46	71	0.8

Table S3. Genes found to be associated with transmissibility in Nebenzahl-Guimaraes *et al.* 2017 in *Mtb* cases collected from the Netherlands. We identified 91 loci that had variation within the Karonga population in these genes, with 52 loci found in only one strain. SNPs and INDELs identified in these genes in more than one Karonga strain are shown, with variants found in both the Karonga population and the Netherlands population italicised. The number of transmitter and non-transmitter Karonga strains harbouring each variant is shown. The number of missing calls in the total strains are also shown. No significant association with transmissibility was found in these genes with the phyC or GWAS analyses. Δ = deletion, + = insertion.

SUPPLEMENTARY METHODS AND RESULTS

The R software, TransPhylo, was used to reconstruct transmission networks, allowing for within host evolution and incomplete sampling. This approach requires the user to define model priors for the underlying transmission reconstruction algorithm. To define appropriate prior values for each parameter we determined initial values based on a review of relevant literature and population level calculations of values from our data. We then conducted a sensitivity analysis, varying specified prior values, on two large transmission cluster of cases linked by up to 50 SNP differences, with the likelihood of transmission links and direction of transmission between cases analysed.

TransPhylo parameters

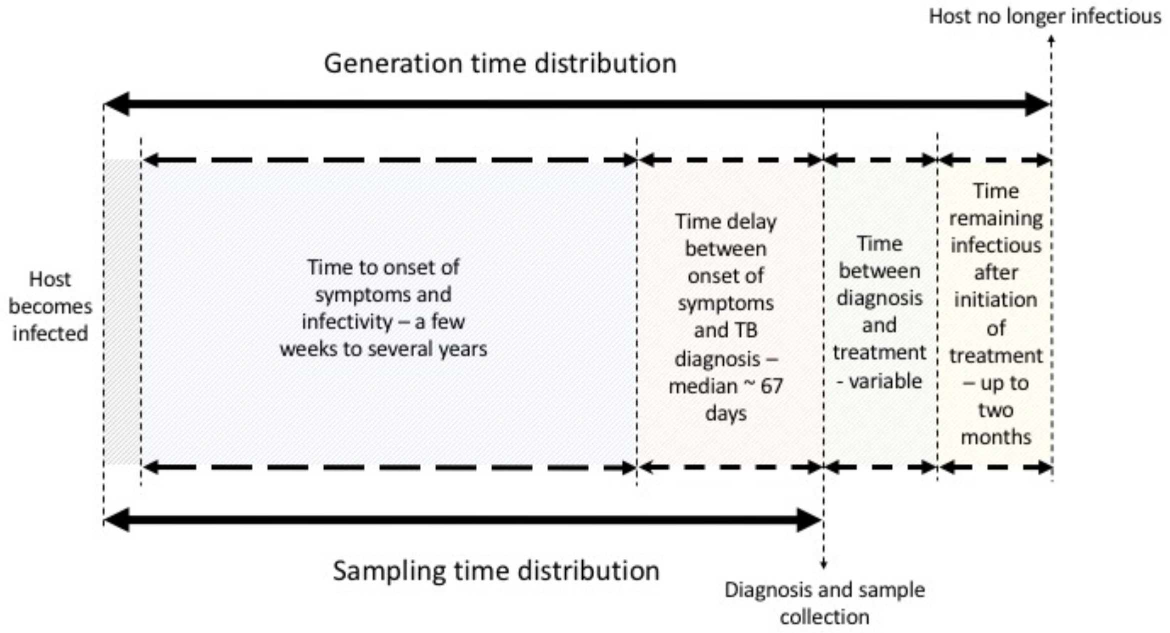

Figure S2. An illustration of the stages of infection within the generation time and sampling time distributions.

Generation time distribution

This is the time between a host becoming infected and infecting another individual (**Figure S2**), modelled as a gamma distribution. This can be highly variable in TB as an individual can develop active, infectious disease relatively quickly after infection in a few weeks or there can be a long period of latency of many years [1,2], though the chances of progression are greatest in the first few years [3]. This rate can also be affected by HIV, which can increase the chances of progression to active disease [4].

The generation time distribution will also include the time that a host may remain infectious and transmit to a recipient while in active treatment, which can be up to two months with effective treatment, though with decreasing infectivity [5,6]. Additionally, it will also include any time between diagnosis and the initiation of treatment. This can be variable depending on whether the patient returns to a healthcare centre for treatment, though the Karonga Prevention Study (KPS) provides support to reduce this risk by including measures such as home visits to patients to encourage them to come for treatment [7].

To account for the variability in the generation time, the gamma distribution choice was modelled to include the chance for a reasonably quick progression to active disease but without penalising transmission trees that include potential long latency in the host. Therefore, prior parameter values chosen were characterised by a relatively rapid rise but with a long tail for latency. We

chose three gamma distribution parameters to test in the sensitivity analysis (**Figure S3**) and posterior distributions were analysed.

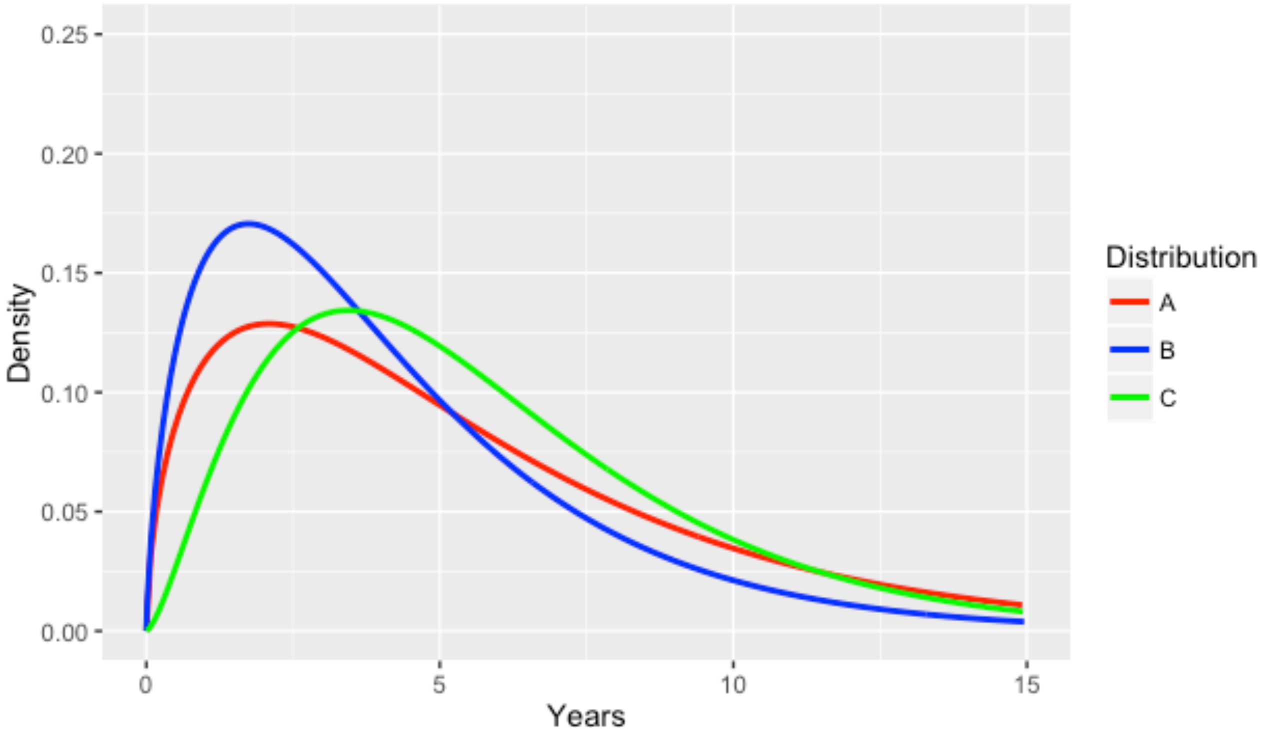

Distribution	Shape	Scale	Rate	Median (Years)	IQR (Years)
**A**	1.6	3.5	0.29	~ 4.5	~ 2.4 – 7.6
**B**	2.2	2.1	0.48	~ 3.9	~ 2.3 – 6.2
**C**	2.5	2.3	0.44	~ 5.0	~ 3.1 – 7.6

Figure S3. Gamma distribution parameter values A, B and C used for the sensitivity analysis.

Sampling time distribution

This is the total time between a host becoming infected and the sample collection. This includes the time between becoming infected to developing symptoms, and the total delay time between a patient becoming symptomatic and receiving a TB diagnosis, including the patient delay time in seeking healthcare and the healthcare system delay in diagnosis (**Figure S2**).

Systematic reviews into delay times in pulmonary TB diagnosis in low-income settings placed the median time from a patient becoming symptomatic to diagnosis to be around 67 days [8,9]; the KPS provides relatively effective detection rates and management of TB [10] and so we would not expect median delay times to exceed this estimate.

In the Karonga Prevention Study (KPS), samples are taken by project staff at hospitals and health centres at the time of diagnosis after screening for symptoms [11], and the first available culture positive sample for each case is used in our analysis. In this population, the sampling time will be similar to the generation time as it will include the all aspects of the generation time apart from the relatively short time remaining infectious after diagnosis (and sample collection) and any delay in the initiation of treatment. Again, the sampling time can be affected by HIV status as these patients may present at healthcare centres more frequently and thus diagnosis may be quicker.

Therefore, we set prior parameters of the sampling time distribution to allow for a large variability in the time to sampling after initial infection. We have used the same distribution as the generation time distribution gamma shape and scale parameter values subject to sensitivity analysis (**Figure S3**).

Reproductive number - R

This is the population-level estimate of the expected number of secondary cases caused by each case. The smear-positive adult TB incidence in the Karonga region has dropped from 124/100,000 per year in the mid-90s to 87/100,000 per year in 2013 [7], so *R* will be < 1. Therefore, the initial value of *R* in the sensitivity analysis was set as 0.8 and updated through MCMC iterations.

Sampling density – π

Over the period of the study there were about 3305 cases of TB, including 3130 pulmonary and 2585 smear positive. After removing isolates that failed QC, have a high probability of mixed infection [12], and that are multiple samples from the same episode of TB in a patient, there were 1857 cases in our dataset. A sampling density (π) of 1 would be equal to the complete sampling of TB cases. Our dataset contains WGS data for 56% of the reported cases of TB in the region (1857 of 3305). This value though does not include undiagnosed cases and cases of transmission from outside the study area. To account for this uncertainty, we have varied the sampling density in the sensitivity analysis with π = 0.4, 0.5 and 0.6, which correspond levels of ~30%, 11% and 0% undiagnosed cases in the population.

Within-host coalescent rate - N_{eg}

If there is no within-host heterogeneity then the transmission tree will correspond directly to the phylogenetic tree, with all transmission events taking place at the same time as phylogenetic coalescent events (nodes of the tree) and all genetic difference between cases evolving at the time of transmission. Incorporating the within-host coalescent rate allows for coalescent events between two cases to be further back in time than the transmission date, i.e. the time to the most recent common ancestor between two cases may be prior to transmission and within a single host. Therefore, there is the possibility that the within-host lineage that transmitted to the secondary case may have been different to the one that is sampled.

This parameter is the rate at which within-host lineages will coalesce (the average time that all lineages within a host (based on the effective population size) can be traced back to their most recent common ancestor), measured in years. The underlying model in TransPhylo employs an extension of Kingsman's coalescent theory [13,14]. The parameter value used corresponds to the product of the effective population size within a host and the generation time. For example, if an organism has a within-host effective population size of 100 and a generation time of 1 day, N_{eg} will equal 100*(1/365) = 0.274 years. In TransPhylo, this parameter is applied as a single value over the population assuming a constant coalescent rate across all samples as well as a bottleneck where only a single within-host lineage is transmitted.

The effective population size, the number of individual bacilli to account for the genetic variation within an organism, will be linked to the mutation rate [15]. The within-host mutation rate for the KPS population has been calculated previously through analysis of SNP differences in longitudinal samples [16], resulting in a rate of 0.45 SNPs per genome per year. A within-host coalescent rate of 1.48 years has been calculated previously for a TB outbreak with a comparable mutation rate (0.48 SNPs per genome per year) [2]. This single value is unlikely to capture the full picture of within-host diversity in all samples and the parameter has been difficult to accurately estimate in simulations with the program [17], with large variation in inferred values. To allow for transmission events before phylogenetic events to not be penalised in inferred transmission trees but owing to the difficulty in estimating this value *a priori*, an initial value of 1.48 was set and updated through MCMC iterations.

Probability thresholds and penalisation for extrapulmonary TB

The output of TransPhylo is a posterior sample set of transmission trees from which the probability of pairwise transmission events between patients can be calculated. Results using the TransPhylo algorithm on a simulated dataset of known transmission events shows that a probability threshold of > 0.5 is sufficient for a specificity rate of 99% and sensitivity rate of 72% [17]. Accordingly, we have chosen a probability threshold of 0.5.

Inferred transmission chains with the transmitter deemed to be a host with extrapulmonary TB were excluded; extrapulmonary TB does not transmit [18,19] so we specified that these cases can only be recipient cases in our analysis.

Sensitivity analysis sampling

The methods used to analyse the sequence data and perform the TransPhylo analysis are described in the methods section of the main paper. A sensitivity analysis was conducted on large transmission clusters, defined as cases linked by up to 50 SNPs, from lineage 2 (n =27) and lineage 3 (n = 37). The generation and sampling time distribution and the sampling density were varied to determine the effect of parameterisation on the inference of transmission links and direction of transmission (**Figure S4)**.

**Run Name**	**Generation and sampling time distribution**	**Sampling density**
A-4	A	0.4
B-4	B	0.4
C-4	C	0.4
A-5	A	0.5
B-5	B	0.5
C-5	C	0.5
A-6	A	0.6
B-6	B	0.6
C-6	C	0.6

Table S4. The generation time and sampling time gamma distributions used in the sensitivity analysis, repeated for two large transmission clusters from lineages 2 and 3. Gamma distributions A, B and C are shown in Figure 2.

Sensitivity analysis results

TransPhylo runs with generation distributions B and C reached convergence over the 10^5 MCMC iterations in the lineage 2 cluster (**Figure S4A**), and all runs reached convergence in the lineage 3 cluster (**Figure S4B**). Considering the posterior generation time distributions for each run, which is the interval for all transmission events including sampled and inferred non-sampled cases with a probability over 0.5 (**Figure S5**), we found that generation time distributions B and C fit the data well. The runs using generation distribution A tended towards a shorter median generation time, which resulted in transmission trees with branches containing an unrealistically high number of inferred, non-sampled hosts with very short transmission times (**Figure S7A and S7D**). Changing the sampling density within each prior generation distribution did not affect the posterior generation time distribution significantly. The reproductive number was updated through the MCMC iterations and the posterior ranged between 1 and 2.5 (**Figure S6)**, thus an initial prior of 1.75 was used for the full analysis and updated through MCMC iterations.

A

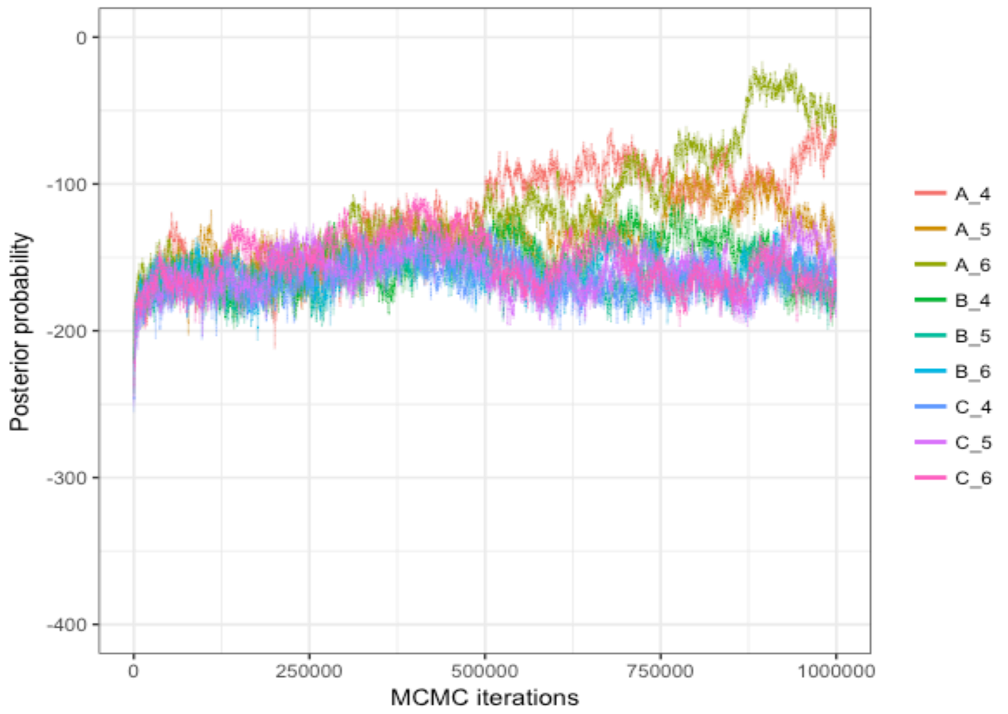

B

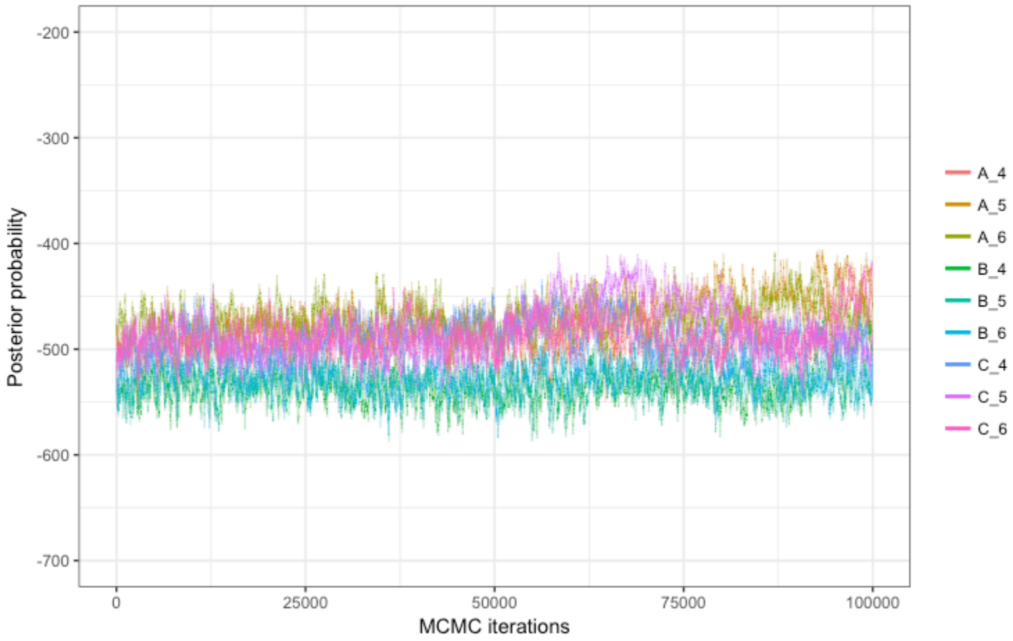

Figure S4. The posterior probability of each TransPhylo run for lineage 2 (A) and lineage 3 (B) clusters after 10^5 MCMC iterations.

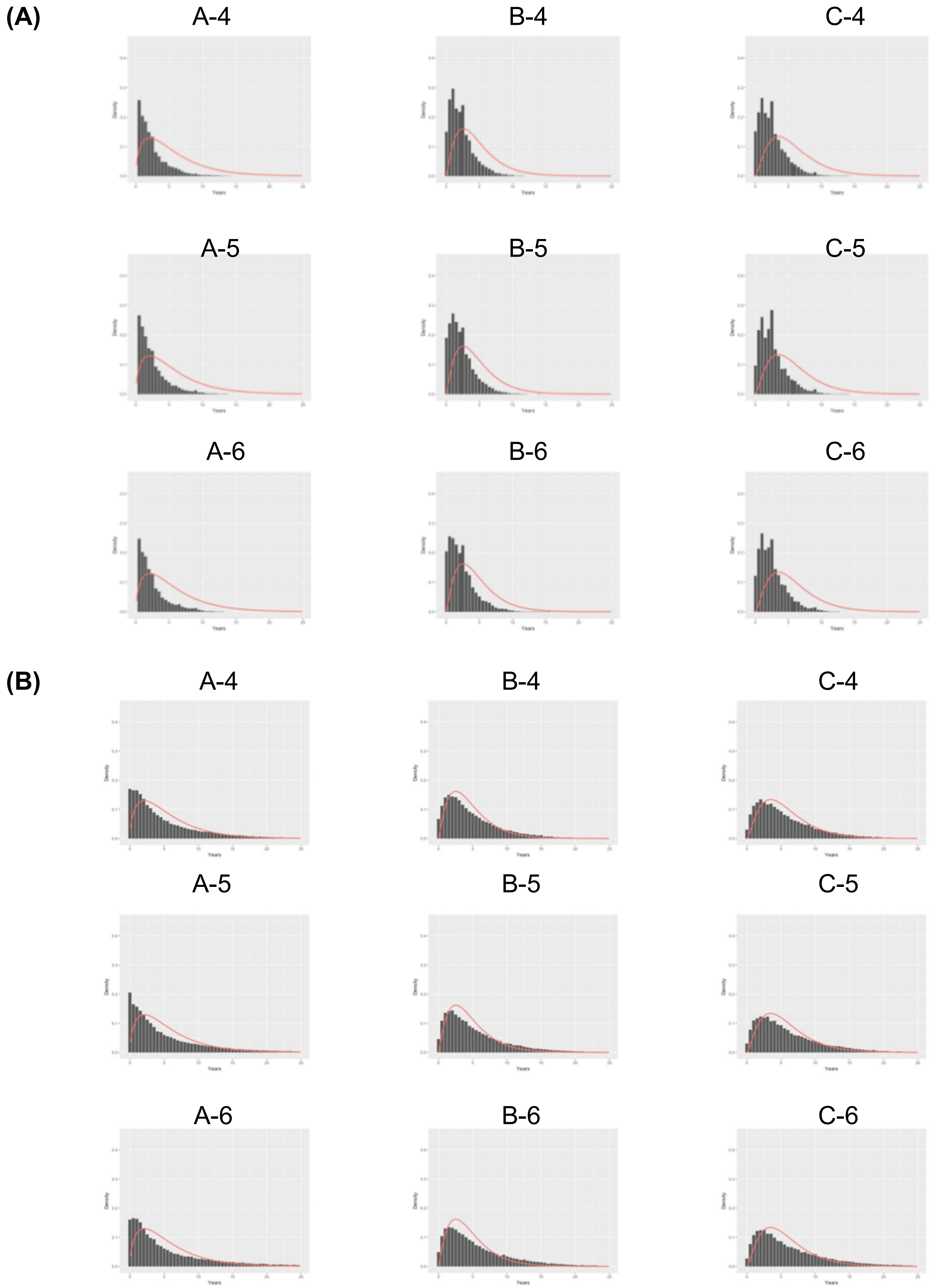

Figure S5. Generation time plots for each run for the lineage 2 cluster (A) and lineage 3 cluster (B), showing the distribution of posterior generation times between all transmission links with a likelihood estimate of > 0.5 (grey bars), along with the prior generation time distribution (red line).

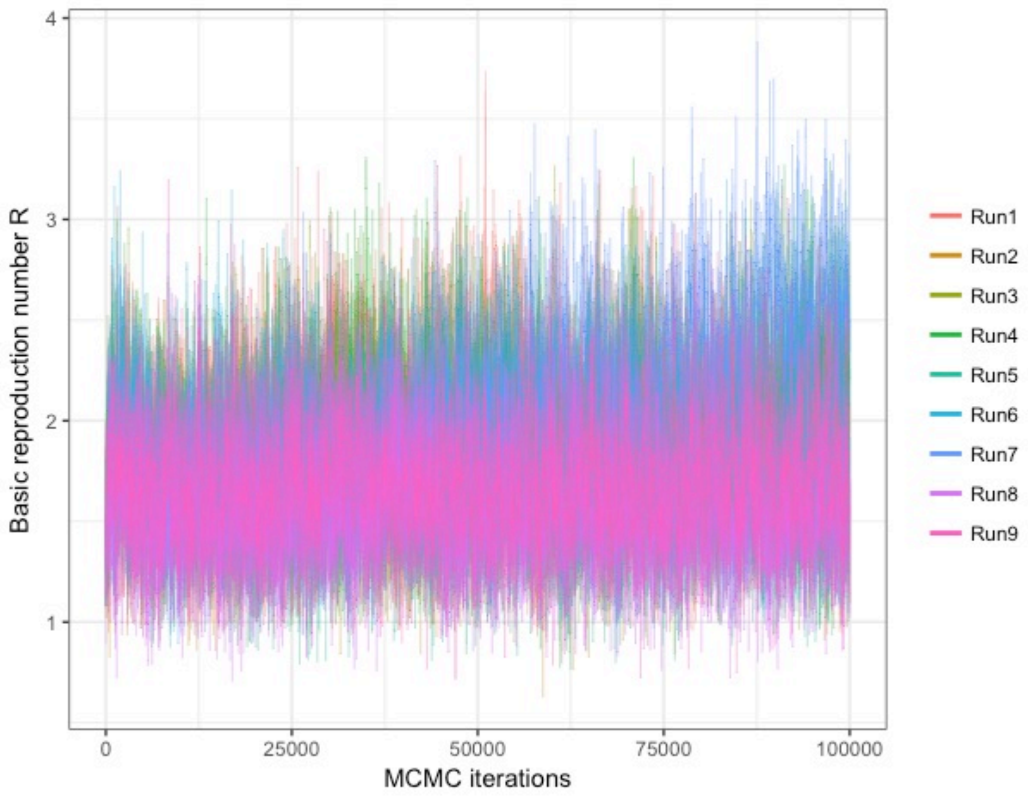

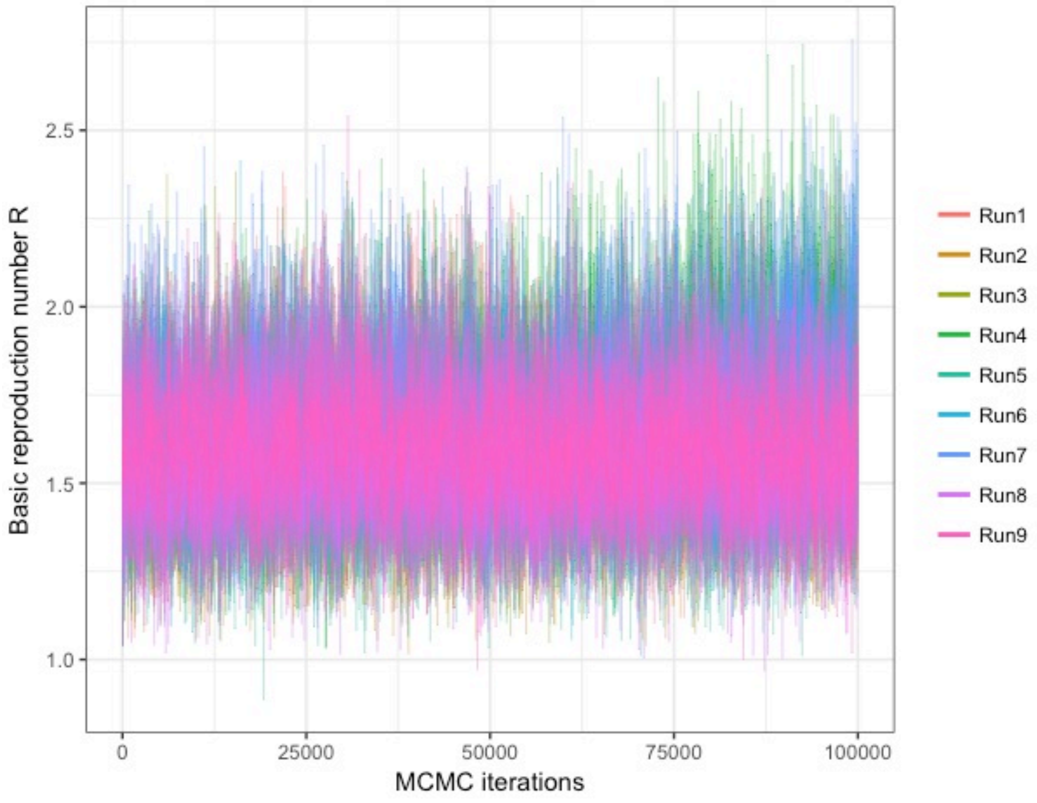

Figure S6. Posterior estimates for the basic reproductive number (*R*) for each run for the lineage 2 cluster (A) and lineage 3 cluster (B).

We found a general agreement in the results of the transmission links inferred by TransPhylo when varying the generation time between distributions B and C, with distribution A leading to varying results (**Tables S5 and S6**). The number of SNPs between sampled cases that were

inferred to be directly linked was between 0 and 2 SNPs in both clusters, with a median of 0 SNP distance (except runs A-4 and A-6).

Inspecting the transmission trees produced by TransPhylo, we found that setting a sampling density of $\pi = 0.4$ led to branches where there were a large number of inferred, non-sampled cases in a short time (illustrated at terminal branches in **Figure S7A and S7D**). While the rates of undiagnosed cases vary across high-incidence countries, it has been reported to be as high as 30-50% [20–22], which would better correspond to a sampling proportion ≤ 0.4. The justification for using a higher sampling density in this study are two-fold. Firstly, the KPS has been working in the Karonga region since 1986 to improve case findings and lower the proportion of undiagnosed TB cases, and this may result in a higher sampling density in our study population than has been reported in other high-incidence areas. Secondly, the starting trees for TransPhylo analysis are subtrees of the whole population that may have different characteristics. Robust surveillance and follow-up of confirmed TB cases with household contact tracing and screening can lead to a higher TB case detection [23] and this may be higher around clustered cases, increasing the within cluster sampling density as compared to the whole population.

Considering only generation distributions B and C and sampling densities 0.5 and 0.6, we found the transmission trees very similar (**Figure S7B and S7C, and Figure S7E and S7F**), and the characterisation of infector strains to be identical in all but one case (ERR181707 in lineage 3, **Table S6**). As it is unlikely that there are no undiagnosed cases within the transmission clusters (corresponding to a sampling density of ~ 0.6), we chose to set the final generation distribution for the full analysis of all clusters in the population as distribution B and a sampling density of 0.5, checking and validating transmission events using INDELs and epidemiologically-linked cases where possible.

	**Lineage 2**		**Lineage 3**	
**Run Name**	**Median**	**Range**	**Median**	**Range**
A-4	0.5	0-2	0	0-1
B-4	0	0-2	0	0-2
C-4	0	0-2	0	0-2
A-5	0	0-2	0	0-2
B-5	0	0-2	0	0-2
C-5	0	0-2	0	0-2
A-6	0.5	0-2	1	0-2
B-6	0	0-2	0	0-2
C-6	0	0-2	0	0-2

Table S5. SNP distance between sampled lineage 2 and lineage 3 cases linked through direct transmission events in with TransPhylo.

Sample	A-4	B-4	C-4	A-5	B-5	C-5	A-6	B-6	C-6	Total
**Lineage 2**										
ERR190368	1	1	1	1	1	1	1	1	1	9
ERR221558	0	0	0	0	0	0	0	0	0	0
ERR221544	0	0	0	0	0	0	0	0	0	0
ERR221574	1	0	0	1	1	1	1	1	1	7
ERR181821	0	0	0	0	0	0	0	0	0	0
ERR245846	1	1	1	1	1	1	1	1	1	9
ERR245680	0	0	0	0	0	0	0	0	0	0

ERR181918	0	0	0	0	0	0	1	0	0	1
ERR221542	1	1	1	1	1	1	1	1	1	9
ERR190401	0	0	0	0	0	0	0	0	0	0
ERR221545	0	0	0	0	0	0	0	0	0	0
ERR212126	1	1	1	1	1	1	1	1	1	9
ERR181916	0	0	0	0	0	0	0	0	0	0
ERR221553	0	0	0	0	0	0	0	0	0	0
ERR212120	0	0	0	0	0	0	0	0	0	0
ERR181769	1	1	1	1	1	1	1	1	1	9
ERR773792	0	0	0	0	0	0	0	0	0	0
ERR323119	0	0	0	0	0	0	0	0	0	0
ERR736808	1	1	1	1	1	1	1	1	1	9
ERR245834	1	1	1	1	1	1	1	1	1	9
ERR245710	0	0	0	0	0	0	0	0	0	0
ERR216958	0	0	0	0	0	0	0	0	0	0
ERR245831	0	0	0	1	0	0	1	0	0	2
ERR245655	1	1	1	1	1	1	1	1	1	9
ERR245723	0	0	0	0	0	0	0	0	0	0
ERR163990	1	1	1	1	1	1	1	1	1	9
ERR245663	0	0	0	0	0	0	0	0	0	0
**Lineage 3**										
ERR176657	0	0	0	0	0	0	0	0	0	**0**
ERR176731	1	1	1	1	1	1	1	1	1	**9**
ERR161145	0	0	0	0	0	0	0	0	0	**0**
ERR181846	0	0	0	0	0	0	0	0	0	**0**
ERR176528	1	1	1	1	1	1	1	1	1	**9**
ERR211997	0	0	0	0	0	0	0	0	0	**0**
ERR176662	0	0	0	0	0	0	0	0	0	**0**
ERR176603	0	0	0	0	0	0	0	0	0	**0**
ERR245704	0	0	0	0	0	0	0	0	0	**0**
ERR190404	1	1	1	1	1	1	1	1	1	**9**
ERR323065	0	0	0	0	0	0	0	0	0	**0**
ERR176464	1	1	1	1	1	1	1	1	1	**9**
ERR037502	0	0	0	0	0	0	0	0	0	**0**
ERR245726	0	0	0	0	0	0	0	0	0	**0**
ERR245646	1	1	1	1	1	1	1	1	1	**9**
ERR181802	0	0	0	0	0	0	0	1	0	**1**
ERR181736	0	0	1	0	1	1	0	1	1	**5**
ERR181864	1	1	1	1	1	1	1	1	1	**9**
ERR245671	0	0	0	0	0	0	0	0	0	**0**
ERR176455	0	0	0	0	0	0	0	0	0	**0**
ERR245796	1	1	1	1	1	1	1	1	1	**9**
ERR036193	0	0	0	0	0	0	0	0	0	**0**

ERR176676	0	0	0	0	0	0	0	0	0	**0**
ERR212027	1	1	1	1	1	1	0	1	1	**8**
ERR037492	1	1	1	1	1	1	1	1	1	**9**
ERR181842	0	0	0	0	0	0	0	0	0	**0**
ERR163974	1	1	1	1	1	1	1	1	1	**9**
ERR161192	1	1	1	1	1	1	1	1	1	**9**
ERR181707	0	0	0	0	0	1	0	1	0	**2**
ERR181683	0	0	0	0	0	0	0	0	0	**0**
ERR216973	0	0	0	0	0	0	0	0	0	**0**
ERR245689	1	1	1	1	1	1	1	1	1	**9**
ERR245732	0	0	0	0	0	0	0	0	0	**0**
ERR245713	0	1	1	1	1	1	1	1	1	**8**
ERR181790	0	0	0	0	0	0	0	0	0	**0**
ERR181804	0	0	0	0	0	0	0	0	0	**0**
ERR245840	0	0	0	0	0	0	0	0	0	**0**
**Total**	**22**	**22**	**23**	**24**	**24**	**25**	**24**	**25**	**25**	

Table S6. Classification by case of infector strains, 1, or non-infector strains, 0, for each run.

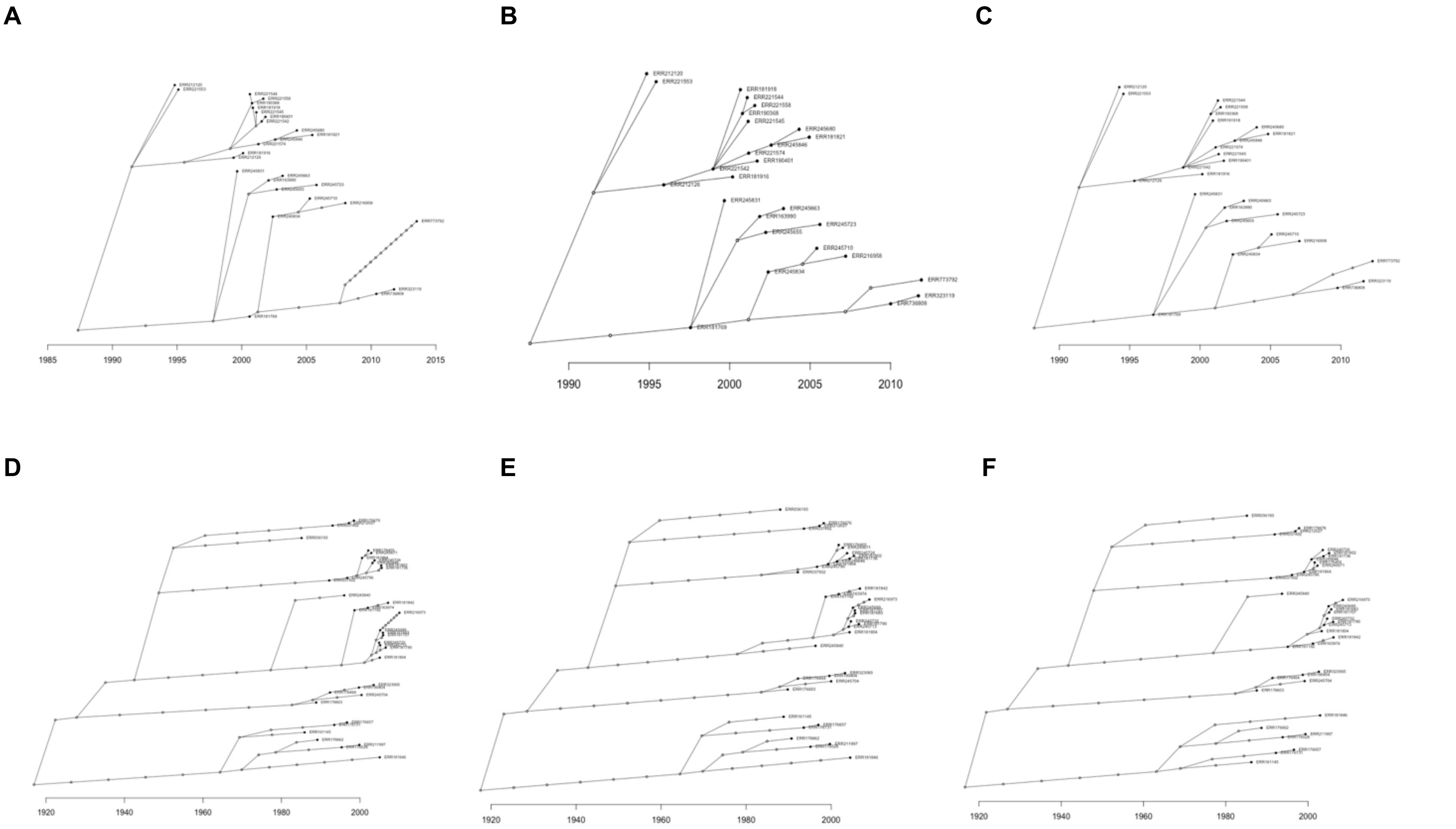

Figure S7. Example transmission trees produced from the TransPhylo output, showing sampled cases (filled points) and inferred, non-sampled cases (unfilled points). **Lineage 2: (A)** Run A-4; distribution A, π = 0.4, **(B)** Run B-5; distribution B, π = 0.5, **(C)** Run C-5; distribution C, π = 0.5, **Lineage 3: (A)** Run A-4; distribution A, π = 0.4, **(B)** Run B-5; distribution B, π = 0.5, **(C)** Run C-5; distribution C, π = 0.5.

References

1. Eldholm, V. *et al.* Impact of HIV co-infection on the evolution and transmission of multidrug-resistant tuberculosis. *Elife* **5**, 1–19 (2016).
2. Didelot, X., Gardy, J. & Colijn, C. Bayesian inference of infectious disease transmission from whole-genome sequence data. *Mol. Biol. Evol.* **31**, 1869–1879 (2014).
3. Andrews, J. R. *et al.* Risk of progression to active tuberculosis following reinfection with Mycobacterium tuberculosis. *Clin. Infect. Dis.* **54**, 784–791 (2012).
4. Pawlowski, A., Jansson, M., Sköld, M., Rottenberg, M. E. & Källenius, G. Tuberculosis and HIV co-infection. *PLoS Pathog.* **8**, (2012).
5. Fitzwater, S. P. *et al.* Prolonged Infectiousness of Tuberculosis Patients in a Directly Observed Therapy Short-Course Program with Standardized Therapy. *Clin. Infect. Dis.* **51**, 371–378 (2010).
6. Ko, Y. *et al.* Duration of pulmonary tuberculosis infectiousness under adequate therapy, as assessed using induced sputum samples. *Tuberc. Respir. Dis. (Seoul).* **80**, 27–34 (2017).
7. Mboma, S. M. *et al.* Control of (Multi)Drug Resistance and Tuberculosis Incidence over 23 Years in the Context of a Well-Supported Tuberculosis Programme in Rural Malawi. *PLoS One* **8**, (2013).
8. Sreeramareddy, C. T., Panduru, K. V., Menten, J. & Van den Ende, J. Time delays in diagnosis of pulmonary tuberculosis: A systematic review of literature. *BMC Infect. Dis.* **9**, 1–10 (2009).
9. Getnet, F., Demissie, M., Assefa, N., Mengistie, B. & Worku, A. Delay in diagnosis of pulmonary tuberculosis in low-and middle-income settings: Systematic review and meta-analysis. *BMC Pulm. Med.* **17**, 1–15 (2017).
10. Crampin, A. C., Glynn, J. R. & Fine, P. E. M. What has Karonga taught us? Tuberculosis studied over three decades. *Int. J. Tuberc. Lung Dis.* **13**, 153–164 (2009).
11. Glynn, J. R. *et al.* Whole genome sequencing shows a low proportion of tuberculosis disease is attributable to known close contacts in rural Malawi. *PLoS One* **10**, 1–12 (2015).
12. Sobkowiak, B. *et al.* Identifying mixed Mycobacterium tuberculosis infections from whole genome sequence data. *BMC Genomics* **19:613**, (2018).
13. Kingman, J. F. . The Coalescent. *Stoch. Process. their Appl.* **13**, 1–7 (1982).
14. Drummond, A. J., Nicholls, G. K., Rodrigo, A. G. & Solomon, W. Estimating mutation parameters, population history and genealogy simultaneously from temporally spaced sequence data. *Genetics* **161**, 1307–1320 (2002).
15. Lanfear, R., Kokko, H. & Eyre-Walker, A. Population size and the rate of evolution. *Trends Ecol. Evol.* **29**, 33–41 (2014).
16. Guerra-Assunção, J. A. *et al.* Large-scale whole genome sequencing of M. tuberculosis provides insights into transmission in a high prevalence area. *Elife* **2015**, 1–17 (2015).
17. Didelot, X., Fraser, C., Gardy, J., Colijn, C. & Malik, H. Genomic infectious disease epidemiology in partially sampled and ongoing outbreaks. *Mol. Biol. Evol.* **34**, 997–1007 (2017).
18. Glynn, J. R. *et al.* Interpreting DNA fingerprint clusters of Mycobacterium tuberculosis. *Int. J. Tuberc. Lung Dis.* **3**, 1055–1060 (1999).
19. Kawamura, I., Kudo, T., Tsukahara, M. & Kurai, H. Infection control for

extrapulmonary tuberculosis at a tertiary care cancer center. *Am. J. Infect. Control* **42**, 1133–1135 (2014).
20. Omar, T. *et al.* Undiagnosed TB in adults dying at home fromnatural causes in a high TB burden setting: A post-mortem study. *Int. J. Tuberc. Lung Dis.* **19**, 1320–1325 (2015).
21. Arega, B., Tilahun, K., Minda, A., Agunie, A. & Mengistu, G. Prevalence rate of undiagnosed tuberculosis in the community in Ethiopia from 2001 to 2014: Systematic review and meta-analysis. *Arch. Public Heal.* **77**, 1–7 (2019).
22. MacPherson, P. *et al.* Disparities in access to diagnosis and care in Blantyre, Malawi, identified through enhanced tuberculosis surveillance and spatial analysis. *BMC Med.* **17**, 1–11 (2019).
23. Lönnroth, K. *et al.* The benefits to communities and individuals of screening for active tuberculosis disease: a systematic review [State of the art series. Case finding/screening. Number 2 in the series]. *Int. J. Tuberc. Lung Dis.* **17**, 432–446 (2013).
